# Understanding Schizophrenia Pathophysiology via fMRI-Based Information Theory and Multiplex Network Analysis

**DOI:** 10.3390/e28010083

**Published:** 2026-01-10

**Authors:** Fabrizio Parente

**Affiliations:** Department of Physiology and Pharmacology, Sapienza University of Rome, P.le A. Moro 5, 00185 Rome, Italy; fabrizio.parente86@gmail.com

**Keywords:** schizophrenia, resting-state fMRI, effective connectivity, causal information transfer, transfer entropy, multiplex network

## Abstract

This work investigates the mechanisms of information transfer underlying causal relationships between brain regions during resting-state conditions in patients with schizophrenia (SCZ). A large fMRI dataset including healthy controls and SCZ patients was analyzed to estimate directed information flow using local Transfer Entropy (TE). Four functional interaction patterns—referred to as rules—were identified between brain regions: activation in the same state (ActS), activation in the opposite state (ActO), turn-off in the same state (TfS), and turn-off in the opposite state (TfO), indicating a dynamics toward converging (Acts/Tfs = S) and diverging (ActO/TfO = O) states of brain regions. These interactions were integrated within a multiplex network framework, in which each rule was represented as a directed network layer. Our results reveal widespread alterations in the functional architecture of SCZ brain networks, particularly affecting schizophrenia-related systems such as bottom-up sensory pathways and associative cortical dynamics. An imbalance between S and O rules was observed, leading to reduced network stability. This shift results in a more randomized functional network organization. These findings provide a mechanistic link between excitation/inhibition (E/I) imbalance and mesoscopic network dysconnectivity, in agreement with previous dynamic functional connectivity and Dynamic Causal Modeling (DCM) studies. Overall, our approach offers an integrated framework for characterizing directed brain communication patterns and psychiatric phenotypes. Future work will focus on systematic comparisons with DCM and other functional connectivity methods.

## 1. Introduction

Revealing functional communication between brain regions from fMRI data is a demanding challenge in computational neuroscience, as reflected by the ingenious and subtle strategies developed over the years. Among these, Dynamic Causal Modeling (DCM), introduced by Friston [1], represents the earliest and most widely adopted framework. DCM enables researchers to infer causal interactions, known as effective connectivity (EC), between brain regions on the basis of hemodynamic modeling [2]. More recently, its application has been extended to resting-state fMRI (rsfMRI), opening new possibilities for network-level investigations [3]. Another classical approach to identifying causal relationships is Granger causality [4,5], although its usefulness is limited by the restrictive statistical assumptions required by the data. To overcome these limitations, non-parametric methods based on information theory—such as Transfer Entropy (TE) [6,7]—have been proposed. Meanwhile, machine-learning techniques and dynamical graph models [8] have also been explored as complementary tools. For an extensive review of causality in fMRI analysis, see Ramsey et al. [9].

In a recent study [10], we introduced a TE-based approach to estimating interactions between brain regions by discretizing BOLD signals into three states (1, 0, and −1) representing supra-, intra-, and sub-threshold activity, respectively. This discretization allows spontaneous events during rest to be detected at a temporal resolution close to that of data acquisition, without substantial information loss [11,12]. Nonetheless, challenges remain, particularly regarding the separation of neural and non-neural BOLD components in spontaneous activity; see Drew [13] for a critical discussion. Within this framework, we described dyadic interactions by tracking state transitions that may occur during information transfer from a region A (state A_t_) to a region B (state B_t_) and its subsequent state (B_t+1_). Among all theoretically possible combinations, only eight exhibited non-random behavior. These are grouped into four “rules”: ActS (activation same), TfS (turn-off same), ActO (activation opposite), and TfO (turn-off opposite). A full methodological description is provided in [10]. Because the resulting interaction matrices can vary across individuals, we employed multiplex-network analysis [14,15] to characterize the structure of the intertwined functional networks. Notably, these rules share key properties with positive and negative functional connectivity (FC) [16]: (1) overlap in modular architecture between ActS and positive-FC networks, and (2) correspondence between central nodes in ActS/positive-FC and ActO/negative-FC networks. Building on these foundations, the present study applies the same methodological framework to resting-state functional images of schizophrenic patients, in order to identify potential markers for clinical and phenotypic characterization.

Schizophrenia is known to involve widespread alterations in brain-network organization, often described as a “disconnection disorder” [17,18,19]. Importantly, this concept does not refer to a simple reduction in connectivity, but rather to heterogeneous and region-specific deviations across the brain. These alterations are thought to arise from disruptions in NMDA-receptor signaling and its modulation by noradrenaline (NE), 5-hydroxytryptamine (5-HT), and dopamine (DA) [17]. Such evidence points to impaired neural plasticity across multiple networks and to failures in corollary discharge mechanisms. Within this context, the dysconnectivity hypothesis provides a neurobiological explanation for core first-rank symptoms of schizophrenia, including loss of agency and experiences of passivity over actions, thoughts, and emotions. Numerous fMRI studies have documented aberrant connectivity within networks such as the default mode network (DMN) [20], salience network (SN) [21], central executive network (CEN) [22], and cerebellum [23]. Inter-network connectivity also appears disrupted, consistent with Menon’s triple-network model [24,25]. Finally, from a network-science perspective [26], the global brain organization of psychotic patients tends to exhibit increased randomness, reflecting reduced segregation and efficiency [27,28], a pattern primarily reported using resting-state FC (rsFC).

Recent methodological developments, including DCM [20,29,30,31] and dynamic functional connectivity (dFC) [32,33,34], have expanded the analytical tools available for characterizing connectivity abnormalities in psychosis. DCM has been particularly informative in schizophrenia research, as it allows for explicit testing of the dysconnectivity hypothesis within models of altered predictive coding [35]. In parallel, dFC captures the moment-to-moment reconfiguration of whole-brain functional networks, enabling the identification of aberrant connectivity states associated with dysconnectivity [36] and enhancing both symptom prediction and diagnostic classification [37]. Extending this perspective, we introduce an analytical framework that estimates directed (effective) connectivity without a priori assumptions, is grounded in information-theoretic principles, and is embedded within a complex-network architecture. This approach enables the characterization of dysconnectivity in psychotic patients by leveraging Transfer Entropy (TE) and multiplex network representations.

## 2. Materials and Methods

### 2.1. Data Collection and Pre-Processing

Anatomical and functional imaging data from 147 subjects, including 72 patients with schizophrenia (SCZ) and 75 healthy subjects (HSs), were obtained from the COBRE (Center for Biomedical Research Excellence) dataset, part of the 1000 Functional Connectomes Classic collection (http://fcon_1000.projects.nitrc.org/indi/retro/cobre.html (accessed on 14 February 2018)). Subjects having a history of neurological disorder, history of intellectual disability, history of severe head trauma (more than 5 min loss of consciousness), and substance use disorder (within the last 12 months) were excluded. To collect medical information, the Structured Clinical Interview for DSM Disorders (SCID) was used. The functional images in the resting-state condition were collected with single-shot full k-space echo-planar imaging (EPI) with ramp sampling correction using the intercommissural line (AC-PC) as a reference (TR: 2 s, TE: 29 ms, matrix size: 64 × 64, 32 slices, and voxel size: 3 × 3 × 4 mm^3^). The anatomical images were acquired with a multi-echo MPRAGE (MEMPR) sequence with the following parameters: TR/TE/TI = 2530/[1.64, 3.5, 5.36, 7.22, 9.08]/900 ms, flip angle = 7°, FOV = 256 × 256 mm, slab thickness = 176 mm, matrix = 256 × 256 × 176, Voxel size =1 × 1 × 1 mm, number of echos = 5, pixel bandwidth =650 Hz, total scan time = 6 min.

Image preprocessing for functional sequences includes realignment and unwarping, normalization to a standard space (EPI image in Montreal Neurological Institute coordinates, MNI), slice timing, and smoothing to a FWHM of 8 × 8 × 8 mm. Anatomical images were normalized in the same space of the functional ones and segmented in the gray matter, white matter and cerebrospinal fluid (CSF). Artifact detection tools (ARTs) were performed to detect outlier scans [38] (framewise displacement above 0.9 mm or global BOLD signal changes above 5 s.d.).

Afterward, noise components from white matter (5 components) and CSF (5 components) and subject-motion parameters (12 components) were adjusted using the anatomical CompCorr method [39]. Finally, a band-pass filtering was applied in the range of 0.008–0.09 Hz: SPM8 (Statistical Parametric Mapping, Wellcome Department of Cognitive Neurology, London, UK).

Throughout the previous steps, the Functional Connectivity Toolbox (CONN) was used on a MATLAB R2018b platform.

### 2.2. Data Extraction and Causal Model

**Figure 1:**
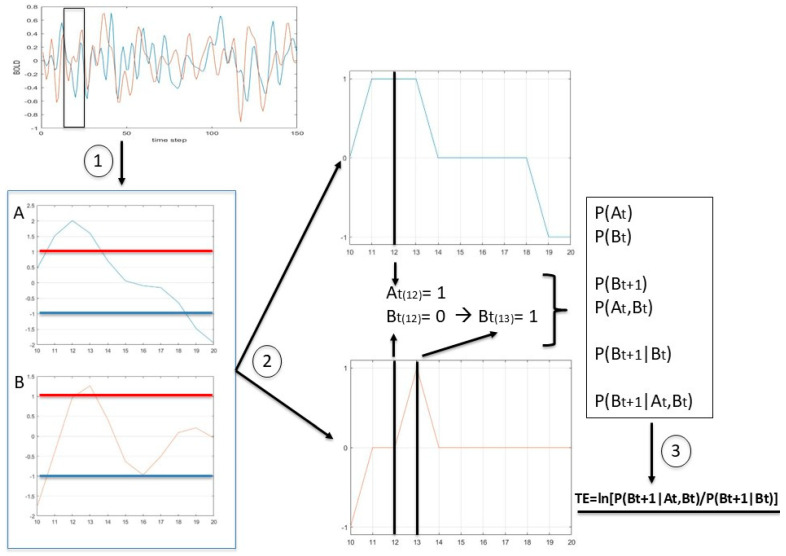
Modeling spontaneous BOLD events. (**Upper left**): BOLD signals of two ROIs, A and B, shown in blue and orange, respectively. (**Lower left**): Z-score–normalized values and detection of spontaneous events above or below the selected threshold (red and blue lines correspond to 1 and −1, respectively). (**Right**): signal discretization into three states (1, 0, and –1). Arrow (1) indicates the zoom on 10 time steps (from 10 to 20) from the total time series and the corresponding Z-score normalization. Arrow (2) indicates the signal discretization: at time step 12, region A is characterized by state (1), and region B by state (0); at time step 13, region B is characterized by state (1). A possible information transfer can be described from region A (in state 1) to region B (which changes state from 0 to 1). By performing this process across all points of the signal, the probability values for each combination of discrete events can be estimated. Arrow (3) illustrates the calculation of TE.

The image voxels of each subject were grouped into 105 Regions Of Interest (ROIs) following the Harvard–Oxford atlas [40] (see the full list of 132 ROIs in Supplementary Materials S1), and the mean time series were extracted. The extracted time series were normalized within each ROI using the Z-score. An increasing threshold was then applied to filter out values above and below specified levels: ±0.25, ±0.50, ±0.75, and ±1. In this way, single events can be selected based on a fraction of the standard deviation, and the selected peaks can subsequently be transformed into the corresponding state values: 1 (above the positive threshold), –1 (below the negative threshold), and 0 (between thresholds). These values correspond to activation (1), deactivation (–1), and null activation (0); see Figure 1. As a matter of fact, these thresholds do not have a particular meaning but were chosen to remain consistent with the previous work [10]. Moreover, in our dataset (see Supplementary Materials S2), this threshold range (0 to ±1) allows us to explore the full spectrum between the minimum and maximum (peak at ±1) amount of information.

To estimate the causal relation between the ROI time series, the TE was calculated for each pair of ROIs by the following equation:

(1) $$T_{A\;\to \;B}=\;\sum P(B_{n+1};\;A_{n}^{\left(k\right)};\;B_{n}^{\left(l\right)})\ln\frac{P\left(B_{n+1}|A_{n}^{\left(k\right)};B_{n}^{\left(l\right)}\right)}{P\left(B_{n+1}|B_{n}^{\left(k\right)}\right)}\;$$

where the state of ROI A and B is specified by *n* = the current time step; *n +* 1 = the following time step; *k* and *l* = number of time steps before the current one (*n*). In our analysis, the temporal repetition (TR) was 2 s, i.e., the time elapsed between two single points within the time series; then, we set the parameters l and k to 1, indicating a delay of 2 s between two sequential states in the time series. The event probability is disentangled among the whole set of possible combinations, and the local TE is calculated (Table 1). Unlike the classical TE, whose components are averaged in a single value, the local TE takes into consideration the weight of each local combination of probability [7]. In such a way, it is possible to detect significant and non-significant components among all combinations; we call these combinations of interactions rules. Since negative local TE values are considered indicative of misinformation transfer [7], we set these values to zero.

To filter out non-significant interaction rules, statistical tests were performed on the TE values for each combination of node states (see [10] for a detailed description). A random model was generated by reshuffling the pre-processed BOLD signals for each subject, altering only the state time sequences while preserving the marginal event frequencies. This model was used to calculate the mean squared error (MSE) between observed and random TE values. The results of this preliminary analysis were consistent with previous work [10], yielding only eight significant interactions. Subsequently, hierarchical clustering [41] was applied to examine the relationships among these interaction rules. Based on this analysis, the interactions were grouped in pairs (see Table 1 for the corresponding conditional probabilities), and only these were used in the subsequent analyses:

1. ActS (activation same): A_n_ (1) + B_n_ (0) → B_n+1_ (1) and A_n_ (−1) + B_n_ (0) → B_n+1_ (−1);
2. TfS (turn off same): A_n_ (1) + B_n_ (−1) → B_n+1_ (0) and A_n_ (−1) + B_n_ (1) → B_n+1_ (0);
3. ActO (activation opposite): A_n_ (1) + B_n_ (0) → B_n+1_ (−1) and A_n_ (−1) + B_n_ (0) → B_n+1_ (1);
4. TfO (Turn off opposite): A_n_ (1) + B_n_ (1) → B_n+1_ (0) and A_n_ (−1) + B_n_ (−1) → B_n+1_ (0).

As can be observed, rules with a similar functional meaning, even if involving different node states, tend to be more closely related to each other. This suggests that dynamical processes operate within different sets of states but may share a common underlying mechanism. Furthermore, by examining the logical consequences of these rules in greater depth, two types of processes can be identified. The first describes an activation process in which a target node (B) transitions from a null activation state (state 0) to an active or deactivated state (1 or −1). The second corresponds to a silencing process, whereby a node transitions from an active or deactivated state to a null activation state. Accordingly, the fundamental processes affecting the target node can be classified as (de)activation—described by ActS and ActO—and turn-off, described by TfS and TfO. Notably, the notion of turn-off is introduced to distinguish processes leading to deactivation (−1) from those resulting in baseline null activation (0). From a broader perspective, in ActS, node A (de)activates node B, bringing it to the same state as A; in TfS, A turns off B when the two nodes are in opposite states. Similarly, ActO causes A to (de)activate B to the state opposite to its own, whereas TfO turns off B when both nodes are in the same state. The first pair of rules (ActS and TfS, hereafter referred to as S) describes a transition of B toward the same state as A (0 → state of A). Conversely, the second pair (ActO and TfO, hereafter referred to as O) characterizes a transition of B toward the opposite state of A (0 → non-A state). This framework enables the description of global system dynamics in terms of uniformity or differentiation, corresponding to converging (S) or diverging (O) states among nodes. A full description of these results is provided in Supplementary Materials S2.

### 2.3. Multiplex Network

The interaction rules define networks characterized by adjacency matrices in which links are not necessarily reciprocal and that are described by a weight (w), indicating the estimated local TE. Thus, a series of weighted and directed networks can be obtained. Since such networks describe interactions between the same brain regions under the same conditions and time, nodes can communicate with each other both within each network and between networks. Thus, we used a multiplex network analysis to study the architecture and dynamics of the interconnected interaction rules between brain regions.

In a multiplex approach [14,15], a system is described by *M* layers, where single networks are associated with single layers α (with *α =* 1, *…*, *M*) of the multiplex network. An adjacency matrix is associated with each layer $W^{\alpha }=\{w_{i,j}^{\alpha }\}$, where $W^{\alpha }$ is the weighted matrix associated with the α layer and $w_{i,j}^{\alpha }$ is the weight from the *i* node (ROI A) to the *j* node (ROI B). A multiplex network can be defined as a vector of adjacency matrices corresponding to layers: *W* = {*W^α^*, …, *W^M^*}. In this way, W includes the complete set of information within the multiplex network while preserving the single-layer structure, allowing the study of network properties derived from a single layer, a subset of N layers (with N < M), or the entire set of layers.

To characterize the overall differences in functional networks between healthy controls and patients, the network density for each rule and each subject was calculated, as well as the total sum of network weights.

### 2.4. Reciprocity and Subgraph Analysis

Reciprocity and subgraph analysis were performed to characterize the structure’s influence on the possible dynamical processes within the networks.

To calculate the reciprocity index (RI), the Squartini et al. [42,43] method was used, which allows for calculating the occurrence of mutual relations within a weighted network. A multiplex extension of the same index, multireciprocity [43], was used to check the probability of links in one layer being reciprocated by the related ones in a different layer within the multiplex. Then, RI values were normalized (nRI) to a null-model (see Section 2.7) to have a range value between [1, −1] indicating a trend of (a) reciprocal connections (nRI > 0), (b) reciprocal avoidance (nRI < 0), and (c) independence (nRI = 0).

**Figure 2:**
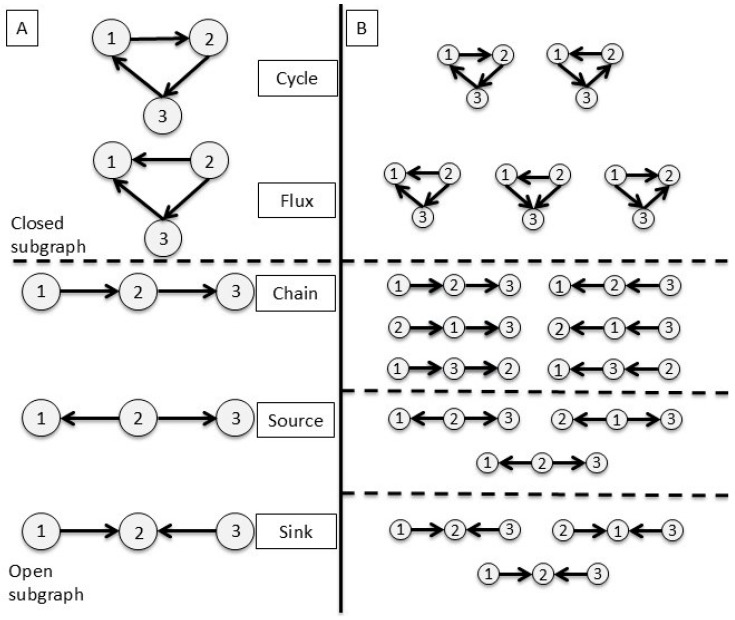
Different configurations of triad subgraphs and their corresponding isomorphisms. Panel (**A**): Closed (**upper**) and open (**bottom**) subgraphs. Closed subgraphs include Cycle, with non-zero in- and out-connections for all nodes, forming a directional cycle; and Flux, with one node showing both in- and out-connections and the other two having only in- or out-links, respectively. Open subgraphs include the following: Chain—three nodes connected by two links in the same direction; Source—two links originating from one node; and Sink—two links converging toward one node. Panel (**B**): Geometrical isomorphisms of each subgraph type. Six Chain isomorphisms arise from the non-symmetrical relations among nodes, as node 2 can be central between nodes 1 and 3 in two opposite configurations. Source and Sink each have three isomorphisms due to the symmetrical relations between peripheral nodes.

A subgraph analysis was performed to characterize motif patterns associated with specific brain regions. According to the graphlet approach [44,45], a subgraph is defined as a combination of in- and out-links among a set of connected nodes, excluding double links. This definition allows the detection of specific geometrical configurations of nodes while avoiding isomorphic structures. In our case, we focus on triads, i.e., combinations of links among three nodes. Specifically, for three nodes, two geometrical patterns are possible in a closed subgraph [45]: Cycle and Flux (see Figure 2). We extended the analysis to open subgraphs, adding three additional patterns, namely the following: (a) Chain—three nodes connected by two links in the same direction; (b) Source—two links originating from one node and directed toward the other two nodes; and (c) Sink—two links directed toward the same node. Figure 2 shows a graphical representation of the complete set of extracted subgraphs.

To quantify the contribution of each subgraph, the intensity index (I) was computed [46]. This measure is defined as the geometric mean of the weights of the links forming a subgraph and characterizes only triads composed of non-zero links. In the case of a cyclic subgraph, for example, in an unweighted and directed network *A*, the number of directed paths returning to the same starting node after *p* steps can be written as: $N=Tr\{A^{p}\}$. For a weighted and directed network *W*, the corresponding intensity is estimated as

$$I=a_{M}Tr\left\{{\left(W^{\left(\frac{1}{p}\right)}\right)}^{p}\right\}$$

where *a_M_* is a combinatorial factor ensuring that each subgraph is counted only once, and *p* was set to 3 (three-node motifs). The direction of links, as well as the absence of links in open triangles, was taken into account using the transpose matrix (*W*^T^) and a “block” matrix (B = [b_ij_], where b_ij_ = 0 when, in the corresponding matrix *W*, either *w_ij_* > 0 or *w_ji_* > 0, and b_ij_ = 1 otherwise), respectively. To characterize triads in a multiplex network, the matrix *W* was decomposed into layer-specific adjacency matrices from layer 1 to *m* (four in our case), i.e., *W* = {*W^α^*, …, *W^M^*}, following the definitions proposed by Battiston et al. [15]. In this framework, a 0th-order triad consists of a subgraph entirely contained within a single layer, whereas 1st- and 2nd-order triads correspond to subgraphs with one link in one layer and the remaining links in a different layer, or with each link belonging to a different layer, respectively. Thus, the intensity value quantifies all combinations of subgraphs characterized by unique, non-isomorphic motifs. This measure was computed for each combination and order for each subject, and its statistical significance was assessed by comparison with a random model (see Section 2.7). The coherence index [46] could also be considered, but it was omitted due to its time-consuming calculation and its limited contribution to our previous results [10].

### 2.5. Centrality

To characterize central nodes within the network, node strength was employed [47]. This index is computed as the sum of the link weights connected to each node. Due to the directional nature of our networks, two centrality indices were calculated: in-strength and out-strength, referring to the links coming and departing each node, respectively. Central nodes are defined as those having a strength value higher than the 90th percentile of the strength distribution, corresponding to the upper quartile. This definition was applied separately for both in-strength and out-strength. Accordingly, in-strength and out-strength central nodes can be interpreted as contributing to the major influx and outflux through the network, respectively. Central nodes were further classified according to their occurrence across layers in the multiplex network: (1) Local central nodes, appearing in a single layer (≤25% of layers); (2) intermediate central nodes, appearing in two or three layers (between 25% and 75% of layers); and (3) multiplex central nodes, appearing in four layers (>75% of layers).

### 2.6. Modularity and Within-/Between-Connectivity

A modularity analysis [48] was performed on the average networks for each group separately. By estimating the Q index, network nodes can be clustered into non-overlapping modules based on their in- and out-connectivity. This analysis was performed on the entire set of rules. To ensure the significance of the partitions, the Q index of the real networks was compared with the same measure computed on a series of randomized models (100 repetitions, see Section 2.7). After identifying the network modules, the anatomical characterization of the resulting sub-networks was performed through a heuristic analysis of the nodes included in each module. Finally, to quantify intra- and inter-modular connectivity, the mean connectivity within and between modules was calculated for each subject in both groups. A systematic comparison of these values between groups was then conducted to detect potential alterations in the functional brain architecture of the SCZ group.

### 2.7. Statistical Analysis

The calculation of the TE measures and the whole set of previous network indexes was performed by an original script (available upon request from one of the authors, F.P.), except for the random models and the modularity analysis, where specific scripts of the Brain Connectivity Toolbox [49] were applied. Each index was calculated for each subject separately. For the randomization procedure, the algorithm proposed by Rubinov and Sporns [50] was employed, which randomizes the weighted directed network while preserving the in-strength distribution. Statistical comparisons were performed using a non-parametric Wilcoxon test, with Bonferroni correction applied when multiple comparisons were required. All analyses were conducted using custom scripts running on the MATLAB R2018b platform.

## 3. Results

### 3.1. Basic Network Metrics

The first analysis focused on the comparison between healthy controls and patients based on the total network weight distribution. The comparison revealed a consistent trend across all rules, showing higher values in patients compared to controls. Additionally, group differences in network density were explored (see Supplementary Materials S3); again, patients exhibited a higher number of connections than controls. Thus, the overall set of patient brain networks appears to be more densely connected. It is worth noting that the differences between groups are on the order of 10^−4^, consistent with the small standard deviation of the measures (see Table 2).

### 3.2. Reciprocity

The entire set of network layers and their interactions appeared significant for (multi)reciprocity compared with the random model in both groups. Each individual layer of the multiplex network showed a negative reciprocity index, indicating asymmetric relationships between brain regions and a preferred direction of interaction. The interactions between layers resulted in a largely symmetric architecture, except for the ActS/TfS and ActO/TfO layer pairs. The comparison between groups revealed a significant increase in negative reciprocity in the TfO matrix of the patient group, indicating greater asymmetry in this network among individuals with schizophrenia. For the complete set of values, see Table 3.

### 3.3. Subgraph Analysis

**Figure 3:**
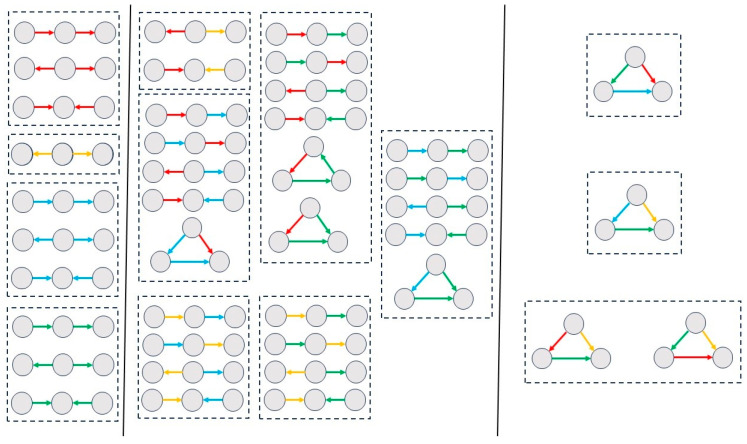
Motifs of the control group. The 0th-, 1st-, and 2nd-order subgraphs are shown in the left, middle, and right panels, respectively. The colored arrows correspond to the following rules: red = ActS; yellow = TfS; blue = ActO; and green = TfO.

**Figure 4:**
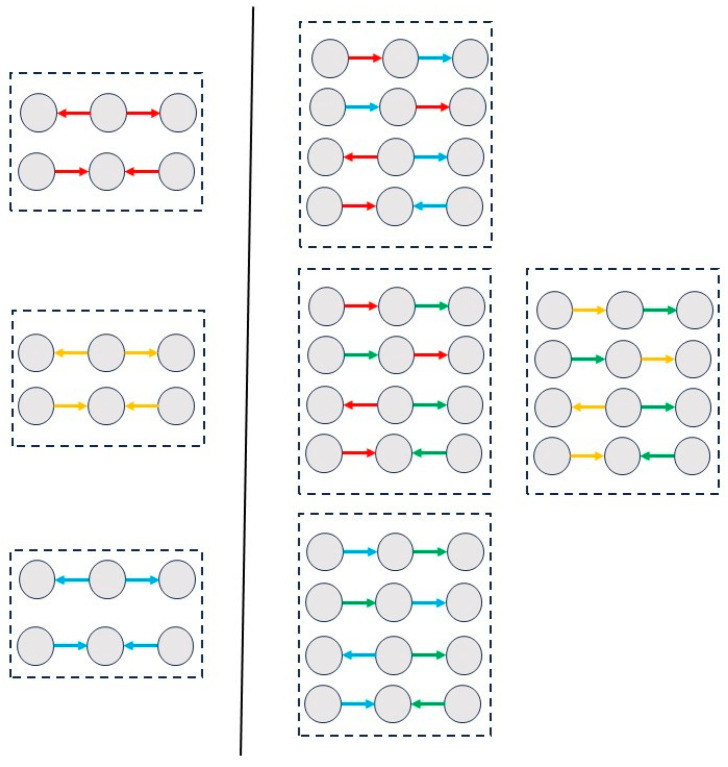
Motifs of the patient group. The 0th- and 1st-order subgraphs are shown in the left and right panels, respectively. No significant results were observed for the 2nd-order subgraphs. The association between colors and rules is shown in Figure 3. A reduced number of motifs was found in this group, with only open triangles detected.

Subgraph analysis revealed distinct sets of subgraph patterns between the groups, as shown in Figure 3 and Figure 4. In both groups, the 0th-order analysis (Figure 3 and Figure 4, left panels) showed significant open triangles compared with the random model. In the 1st-order analysis, closed triangles appeared only in the HS group (Figure 3, middle panel). Specifically, three flows were observed, composed of the following: (a) ActS and ActO rules, (b) ActS and TfO rules, and (c) ActO and TfO rules; additionally, a cycle consisting of ActS and TfO rules was detected. In the 2nd-order analysis (Figure 3, right panel), four additional subgraphs were detected only in the HS group: two sharing the same rules (ActS, TfS, and TfO), and two others composed of the following combinations of rules: (a) ActS, ActO, and TfO, and (b) ActO, TfS, and TfO. In the SCZ group, neither closed triangles in the first-order motifs nor subgraphs in the second-order motifs were detected.

A systematic statistical comparison between groups was performed for each possible subgraph (see Supplementary Materials S4, Tables S3–S5 for the 0th-, 1st-, and 2nd-order subgraphs, respectively). The analysis revealed a general trend of increased intensity values in the SCZ group across almost all orders and subgraphs. Open subgraphs containing the rules ActO and TfO in the zeroth-order motifs were not significant, nor was the first-order open triangle formed by one connection with ActS and the other with ActO or TfO.

Overall, the SCZ group exhibited a reduced number of non-random motifs and a reduced complexity of subgraph types (i.e., only open triangles), while showing higher intensity values compared with the HS group.

### 3.4. Centrality Analysis

**Figure 5:**
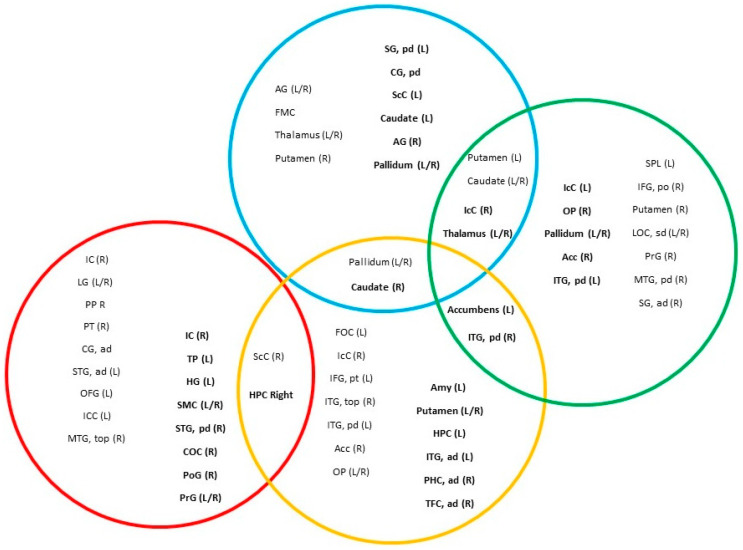
Central nodes of the control group. The colored circles correspond to the rules: red = ActS; yellow = TfS; blue = ActO; and green = TfO. Within each circle, the brain regions acting as local central nodes are reported, while intermediate multiplex central nodes are shown in the intersections. Out-strength central nodes are highlighted in bold. Abbreviations: IC: insular cortex; LG: lingual gyrus; PP: planum polare; PT: planum temporale; CG, ad: cingulate gyrus, anterior division; STG, ad: superior temporal gyrus, anterior division; OFG: occipital fusiform gyrus; IcC: intracalcarine cortex; MTG, top: middle temporal gyrus, temporo-occipital part; ScC: supracalcarine cortex; FOC: frontal operculum cortex; IFG, pt: inferior frontal gyrus, pars triangularis; ITG, top: inferior temporal gyrus, temporo-occipital part; ITG, pd: inferior temporal gyrus, posterior division; Acc: accumbens; OP: occipital pole; AG: angular gyrus; FMC: frontal medial cortex; SPL: superior parietal lobule; IFG, po: inferior frontal gyrus, pars opercularis; LOC, sd: lateral occipital cortex, superior division; PrG: precentral gyrus; MTG, pd: middle temporal gyrus, posterior division; SG, ad: supramarginal gyrus, anterior division; TP: temporal pole; HG: Heschl’s gyrus; SMC: supplementary motor cortex; STG, pd: superior temporal gyrus, posterior division; COC: central opercular cortex.

A different pattern of central nodes can be observed across the layers of the multiplex network in both groups. In the HS group (see Figure 5 and Supplementary Materials S5, Table S6), the in-strength hubs of the ActS rule were primarily located in sensory and sensory-processing areas of the occipital and temporal cortices, while out-strength hubs were found in primary somatosensory and motor cortices, medial and lateral temporal structures, and the insula. Under the TfS rule, in-strength hubs included the occipital lobe, lateral prefrontal cortex, and pallidum, whereas out-strength hubs were mainly subcortical (amygdala, putamen, caudate) and temporal regions (hippocampus, parahippocampus, fusiform gyrus). For the ActO rule, both in- and out-strength hubs involved the angular gyrus and subcortical regions (thalamus, pallidum, caudate), with additional in-strength nodes in the putamen and medial frontal cortex, and out-strength nodes in the supramarginal gyrus, posterior cingulate, and primary visual cortex. The TfO rule showed sparse in-strength hubs in fronto-parietal, occipital, temporal, and basal ganglia regions, while out-strength hubs were mainly subcortical and occipito-temporal.

Subcortical regions emerged as the main interlayer hubs, appearing across three layers (TfS, ActO, TfO). Other intermediate hubs included the supracalcarine cortex, hippocampus, inferior temporal gyrus, and intracalcarine cortex. Overall, shared nodes between rules were as follows: ActS/TfS (one in-strength, one out-strength), TfS/ActO (two in-strength, one out-strength), TfS/TfO (two out-strength), and ActO/TfO (three in-strength, three out-strength).

**Figure 6:**
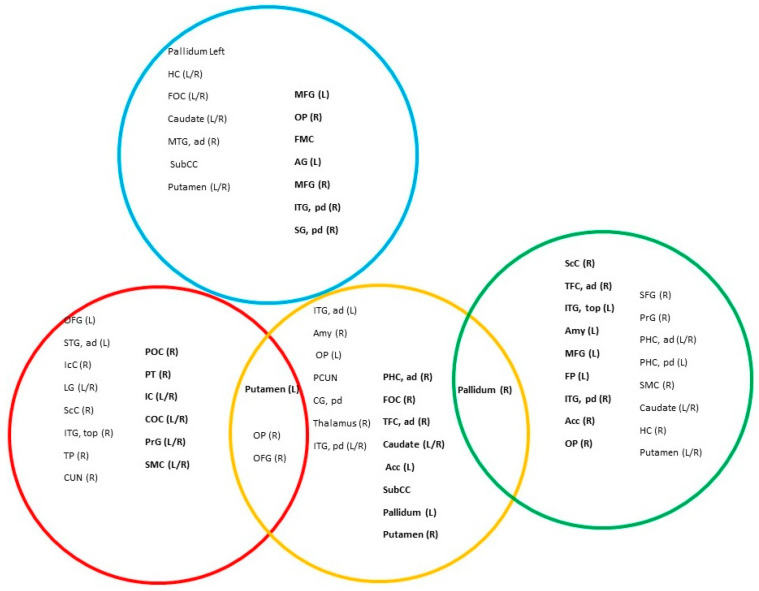
Central nodes of the patient group. The colored circles correspond to the rules: red = ActS; yellow = TfS; blue = ActO; and green = TfO. Within each circle, the brain regions acting as local central nodes are reported, while the intersections indicate intermediate multiplex central nodes. Out-strength central nodes are highlighted in bold. Notably, the patient group shows fewer intersections among circles, suggesting a decreased tendency toward intermediate multiplex central nodes. Abbreviations: OFG: occipital fusiform gyrus; STG, ad: superior temporal gyrus, anterior division; IcC: intracalcarine cortex; LG: lingual gyrus; ScC: supracalcarine cortex; ITG, top: inferior temporal gyrus, temporo-occipital part; TP: temporal pole; CUN: cuneus; OP: occipital pole; POC: parietal operculum cortex; PT: planum temporale; IC: insular cortex; COC: central opercular cortex; PrG: precentral gyrus; SMC: supplementary motor cortex; ITG, ad: inferior temporal gyrus, anterior division; Amy: amygdala; PCUN: precuneus; CG, pd: cingulate gyrus, posterior division; ITG, pd: inferior temporal gyrus, posterior division; PHC, ad: parahippocampal gyrus, anterior division; PHC, pd: parahippocampal gyrus, posterior division; FOC: frontal orbital cortex; TFC, ad: temporal fusiform cortex, anterior division; Acc: accumbens; SubCC: subcallosal cortex; HC: hippocampus; FOC: frontal operculum cortex; MTG, ad: middle temporal gyrus, anterior division; MFG: middle frontal gyrus; FMC: frontal medial cortex; AG: angular gyrus; SG, pd: supramarginal gyrus, posterior division; SFG: superior frontal gyrus; PrG: precentral gyrus; FP: frontal pole.

In the patient group (see Figure 6, Section 5, and Supplementary Materials S5, Table S7), a pattern similar to that observed in healthy subjects emerges for the ActS rule, with in-strength central nodes located in the occipital and temporal lobes, and out-strength central nodes in the somatosensory cortex. Similarly, the TfS rule shows central nodes in temporal and subcortical brain regions, with basal ganglia nodes predominating as out-strength hubs. Under the ActO rule, in-strength central nodes are found in subcortical regions, the hippocampus, the frontal operculum cortex, and the middle temporal gyrus. Out-strength hubs include a more distributed set of regions spanning the medial and lateral frontal cortices, superior parietal areas, occipital regions, temporal lobe, and posterior cingulate cortex, with many of these regions belonging to the DMN. For the TfO rule, in-strength central nodes are located in the frontal lobe (superior frontal gyrus, precentral gyrus, and supplementary motor cortex), basal ganglia, hippocampus, and parahippocampal cortex. Out-strength central nodes are mainly found in the occipital lobe (supracalcarine cortex and occipital pole), temporal lobe (inferior temporal and fusiform gyri), frontal lobe (middle frontal gyrus and frontal pole), and subcortical regions (accumbens, amygdala, and pallidum).

Finally, for both the ActS and TfS rules, the fusiform gyrus and occipital pole appear as in-strength intermediate multiplex central nodes, while the putamen serves as an out-strength intermediate hub.

The statistical comparison between groups (Table 4) reveals an overall increase in strength values in the patient group. Regarding individual rules, ActS shows a limited effect, with increased values observed only in the supplementary motor areas (in-strength) and the intracalcarine cortex (out-strength) in the SCZ group. Similar results are seen for the TfS rule, with higher values in regions of the visual processing network (fusiform and lingual gyri), as well as in the insula, putamen, supramarginal gyrus, and planum polare. More pronounced differences are evident in the ActO and TfO rules. For ActO, increased in-strength is found in the lateral prefrontal cortex (pars orbitalis and pars opercularis), insula, parahippocampal cortex, planum polare, and subcallosal cortex. The out-strength pattern shows a broader distribution, primarily involving the temporal lobe (superior, middle, and inferior temporal gyrus), along with the superior frontal gyrus, paracingulate cortex, precuneus, fusiform gyrus, and hippocampus. For the TfO rule, higher in-strength is observed in the insula, hippocampus, parahippocampal cortex, anterior cingulate cortex, putamen, as well as in temporal regions (inferior temporal gyrus, temporal pole, and fusiform gyrus) and lateral prefrontal areas. The out-strength pattern largely mirrors that of ActO, with predominant effects in the temporal lobe (planum temporale, fusiform, middle, and inferior temporal gyri) and additional involvement of the frontal pole, postcentral gyrus, lateral occipital cortex, posterior cingulate cortex, and amygdala.

Overall, in-strength measures are characterized by increased values in the insula, frontal operculum, and parahippocampal cortex, consistent findings across both ActO and TfO rules. In contrast, different portions of the fusiform, middle, and inferior temporal gyri appear as the main out-strength regions affected in the patient group.

### 3.5. Modularity and Network Architecture

**Figure 7:**
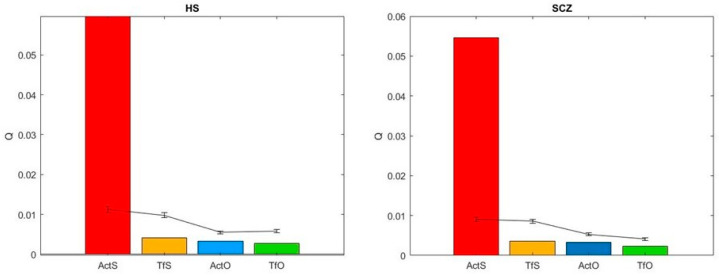
Estimated Q-values for each rule in both groups. Only the ActS rule shows a significantly greater value compared to the random models. The black lines are the random models. Bar plot colors denote the interaction rules: red = ActS; yellow = TfS; blue = ActO; green = TfO.

The Q-index was significantly higher than the random control in both groups for the ActS rule only (see Figure 7). As shown in Table 5, four main clusters were identified in the HS group. The first cluster included sensory (excluding visual) and motor-related brain regions, as well as nodes of the salience and attention networks (module 1). The second cluster overlapped with the visual network (module 2). The third corresponded to most areas of the limbic network (module 3). The fourth comprised a sparse set of regions belonging to the CEN, the DMN, and the basal ganglia (BG). An iterative application of the modularity algorithm on this last cluster revealed a significant hierarchical subdivision into three additional modules: one including only the BG (module 4), a second representing the CEN (module 5), and a third encompassing the DMN along with other scattered cortical regions. Further splitting of this third cluster yielded another significant parcellation, resulting in a distinct DMN module (module 6) and a set of fronto-temporo-occipital cortices (module 7). No further significant subdivisions were observed after comparison with randomized models. Asymmetric lateralization was observed within the modules: the left and right frontal operculum were included in modules 5 and 1, respectively, while the left and right anterior inferior temporal gyrus were included in modules 7 and 3, respectively.

The SCZ group exhibited only minimal heuristic differences compared to the HS group. Specifically, three main modules were identified: the first two corresponded to the same brain regions as in the HS group, except for the posterior supramarginal gyrus, which was lateralized solely to the right in module 1. The third module was again significantly subdivided into two modules, covering a sparse ensemble of regions. These two modules were further divided, though no additional significant segregation was observed. The first was subdivided into three modules: one covering the limbic network (module 3), one including the BG (module 4), and one corresponding to the DMN (module 6). The second was split into two modules: one representing the CEN (module 5) and another overlapping with module 7, already identified in the HS group. Asymmetric lateralization was also observed in the SCZ group, with the left and right posterior SMG and frontal operculum assigned to modules 5 and 1, respectively. Minimal anatomical differences between groups were noted in the nodes included in each module: the anterior inferior temporal gyrus was included in the limbic module (3) for both hemispheres in the SCZ group, whereas the posterior SMG was divided between module 1 (right) and module 5 (left).

A direct statistical comparison between groups showed that, in the SCZ group, within-module connectivity was increased across all modules except the BG module (module 4). The ActS, ActO, and TfO rules showed increased connectivity in modules 1 and 2, whereas ActO and TfO were increased in modules 4, 5, 6, and 7. Most of the increased between-module connectivity patterns in the patient group were observed in the out- and in-strength connections from modules 1 and 2 toward all other modules, respectively. Another prominent pattern involved the ActS, ActO, and TfS rules, with increased connectivity from several module sets toward the BG (module 4). See Table 6 for a detailed characterization of the altered connectivity within- and between-modules.

## 4. Discussion

In this paper, we propose a dynamic characterization of the functional brain network using a data-driven approach to identify phenotypic differences associated with schizophrenia. TE [6,7] was used as an estimate of EC. According to a previous study [10], TE enables the characterization of both non-random connectivity and well-defined network architectures, suggesting a possible model-based description of functional processes in the brain. The same analytical pipeline described in [10] was employed, based on a discrete approach to time-series analysis. Accordingly, the continuous values of the BOLD signal were converted into a series of finite states by filtering the Z-normalized time series. As reported in earlier studies [11,12], this procedure aims to detect possible spontaneous events in brain signal fluctuations that are closer to the temporal resolution of signal acquisition. We identified four types of interactions between brain areas (ActS, TfS, ActO, and TfO) that significantly differed from random controls and were organized in a hierarchical structure. These brain interactions can be understood as manifestations of a general mechanism involving activation, deactivation, and turn-off processes. To characterize the brain networks of both controls and patients and to estimate potential differences, we employed a multiplex network approach. For each interaction rule, a matrix was computed and subsequently combined into a multidimensional matrix for each subject, corresponding to a multiplex directed network.

### 4.1. Networks of Healthy Controls

The results obtained in the health control group appear to be partially consistent with our earlier findings [10], and a critical discussion is provided below. Centrality measures indicate distinct hub patterns across the different interaction rules:

1. ActS: Central brain regions are located within the sensorimotor and sensory pathways;
2. ActO: In the basal ganglia and default mode network (DMN) regions;
3. TfS: In the basal ganglia and temporal regions;
4. TfO: In the basal ganglia and a sparse set of cortical brain regions.

As observed in the previous analysis [10], the basal ganglia emerge as the regions with the greatest overlap across rules, particularly shared by the turn-off rules and ActO. Notably, the central nodes identified in the ActS rule overlap with the most synchronized brain regions found using an alternative functional connectivity estimation approach [51]. This would not be surprising, as the ActS rule characterizes a direct activation (or deactivation) of a target node into the same state as the source node, potentially describing a synchronization mechanism.

The reciprocity results appear consistent with our previous paper [10], indicating non-symmetrical interactions within the intra-layer turn-off rules. Moreover, the inter-layer interactions also closely match our earlier findings:

1. symmetrical interactions of ActS/TfO, ActO/TfS, and TfS/TfO;
2. asymmetrical interactions of ActS/TfS and ActO/TfO.

**Figure 8:**
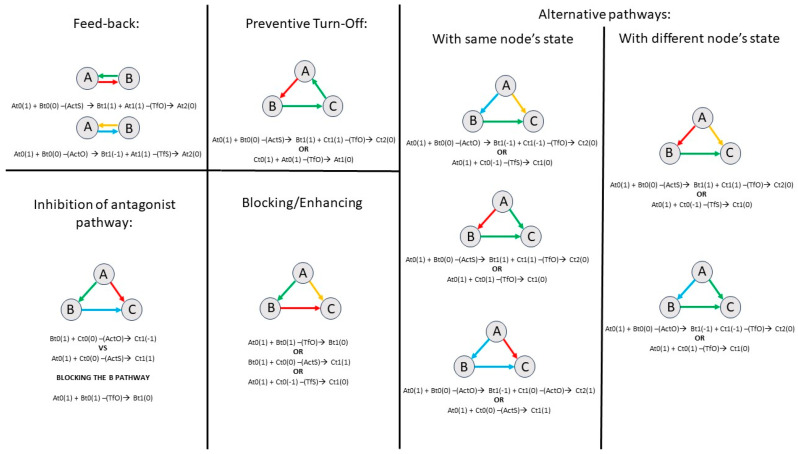
Possible dynamic process for each motif found in the networks. Several mechanisms can be inferred from the dynamics between nodes: (a) feed-back: node A (de-)activates node B, which consequently turns off node A; (b) inhibition of antagonist pathway: nodes A and B exhibit antagonistic dynamics toward node C (ActS vs. ActO), where node A turns off node B via TfS to resolve the conflict; (c) preventive turn-off: node A de-activates node B which in turn turns off node C, thereby preventing the turning off of node A; (d) blocking/enhancing: node A can stop or enhance the dynamics between nodes B and C by turning off either node B or node C, respectively; and (e) alternative pathways: node A influences node C either indirectly through node B (indirect path) or directly on node C (direct path). Two possible scenarios arise: in one scenario, nodes B and C share the same state; in the other, they have different states. The equations under the picture refer to the time changing node’s state, *N_ti_* indicates a given node *N* at time *i*, -(rule)→ indicates the rule operating in that information transfer. Arrow colors denote the interaction rules: red = ActS; yellow = TfS; blue = ActO; and green = TfO.

Interestingly, the first two symmetric interactions can be interpreted as a feedback mechanism, where a node (A) that has (de-)activated another node (B) can itself be turned off by node B (see Figure 8, upper and left panels for a schematic representation of this feedback mechanism). Other dynamic processes can be explored through subgraph analysis. As a preliminary observation, we found that most open triangles differ significantly from random patterns. These substructures have not been investigated in previous studies [10], suggesting a likely central role of these subgraphs in information flow within the brain system. In this study, fewer closed triangles were characterized compared to our previous work [10]. One possible explanation for this discrepancy is that the dataset used here involved a different repetition time (RT) in scan acquisition, making the dynamic processes captured by subgraph analysis more sensitive to temporal sampling differences. The subgraph patterns identified in the present work appear to be characterized by particular dynamic mechanisms, as illustrated in Figure 8. It cannot be excluded that different motifs may emerge at different sampling frequencies; therefore, further analyses employing various temporal sampling rates of the BOLD signal are needed to explore this hypothesis.

In general, the dual mechanisms of stabilization (ActS and TfS) and destabilization (ActO and TfO) can be understood as dynamic modulations of node states associated with decreased or increased variability between brain regions, respectively. It is therefore plausible to hypothesize that the ActS rule tends to stabilize connections between different brain regions within the same subnetwork. Supporting this view, modularity analysis revealed a significant modular architecture only for the ActS rule, reinforcing its potential role in the dynamic reverberation of intra-module connections. Conversely, the ActO rule appears to counterbalance this process, thus characterizing the architecture of negative (or anti-phase) functional connectivity [16]. Finally, it is reasonable to speculate that the turn-off rules may play a specific role in modulating synaptic excitability, reflecting the brain system’s adaptation to internal and external changes, such as gain modulation [52]. Further discussion will follow in the next paragraphs, after addressing the altered network in schizophrenic patients.

### 4.2. Dysconnectivity of Brain Functional Connectivity in Schizophrenia

As a general framework, the estimation of brain functional causal interactions allows us to validate the pathophysiological model of schizophrenia. Among various connectivity measures, Dynamic Causal Modeling (DCM) [1] aligns most closely with our TE analysis. However, several advantages emerge when using a TE-based approach: (1) the ability to characterize a directed whole-brain network and study its specific topological features, and (2) the estimation of causal interactions in resting-state conditions. In this context, recent advances in DCM allow such analyses even in the absence of external stimuli [3]; however, unlike DCM, our method estimates directed connections without explicit a priori assumptions. Taken together, these considerations support a critical comparison between our findings and those obtained using DCM methods.

**Figure 9:**
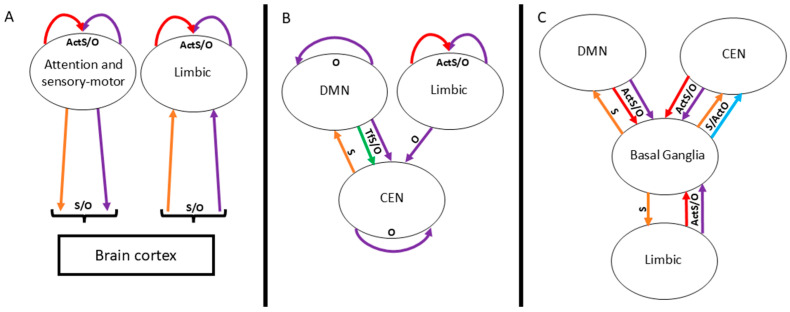
Main alterations of the functional architecture of the SCZ brain network. Panel (**A**): Increased connectivity across all rules, with outgoing connections primarily targeting the attentional/sensorimotor network and incoming connections mainly involving the limbic network. Panel (**B**): Opposite alterations between DMN and CEN, with the limbic network showing enhanced O-rule (ActO/TfO) influence on CEN. Panel (**C**): Basal ganglia as a central hub mediating interactions among DMN, CEN, and limbic networks. Overall, schizophrenia-related network alterations can be summarized as (1) increased weight of sensory pathways across the brain, (2) disrupted dynamic balance within and between associative networks, leading to reduced node-state consensus, and (3) a potential central role of the basal ganglia, consistent with the CSPTC model; see the text for details. Arrow colors denote the interaction rules: red = ActS; yellow = TfS; blue = ActO; green = TfO; orange = S (ActS/TfS); and purple = O (ActO/TfO). CEN = central executive network and DMN = default mode network.

Our results revealed that, in all rules, both connection weight and density were increased in the SCZ group. At the level of individual node connectivity, distinct patterns of brain regions with elevated TE values emerged in patients, with most differences observed in out-strength measures, and particularly in the ActO and TfO rules. Additionally, the overlap of nodes across different layers of the network was reduced in the SCZ group, suggesting a less integrated and less complex pattern of interactions between rules. Regarding the ActS rule, the SMA showed significant increases of the in-strength in the patient group. This region has previously been implicated in schizophrenia using DCM [29,30], with evidence of reduced auto-inhibitory connectivity [29] and overall increased effective connectivity [30]. The SMA is associated with several cognitive deficits in schizophrenia [53], making our findings consistent with prior observations. Moreover, the ActS rule showed increased out-strength in the intracalcarine cortex, indicating selective enhancement of bottom-up sensory pathways [35]. For the TfS rule, no differences were observed in in-strength connections, while out-strength was increased in secondary sensory processing and salience networks, as well as in sparse cortical and subcortical regions. This pattern may reflect enhanced excitability of target regions, favoring a persistent activation state across the network. Overall, these results suggest an anomalous dual mechanism of sensory activation and deactivation, affecting sensory processing, salience (insula), and motor pathways (putamen), with an enhanced bottom-up influence consistent with prior studies in schizophrenia [35]. In the between-module connectivity analysis, the attention/sensorimotor and limbic networks showed the most pronounced alterations, with increased out- and in-strength values, respectively, across all brain networks and rule sets (Figure 9A). This finding highlights the sensorimotor cortex’s role in perceptual alterations [53] and confirms the limbic network’s involvement in intra- and inter-module dysconnectivity in psychiatric disorders [54]. The in- and out-strength of the ActO and TfO rules revealed a distinctive pattern of altered brain regions. Most of the affected nodes were located in association cortices and modulatory regions, including the temporal lobe, DMN, frontal lobe, and insula. ActO and TfO appear most impacted in SCZ, possibly reflecting increased variability in node states, network instability, and heightened system noise: a potential marker of schizophrenia [31]. As a matter of fact, decreased stable activations and increased system noise have been linked to less efficient network topology in rsFC studies of schizophrenia [27,28]. Although segregation and integration properties of TE networks were not directly assessed in this work, the reduced subgraph complexity found in the patients’ group likely reflects impaired network structure and altered dynamic interactions, suggesting a shift toward more random network organization [28,55]. Interestingly, despite fewer significant patterns of subgraphs, their intensity increased in patients, consistent with a general disinhibition of connections that may reduce network stability and organization. Future studies should address this point more explicitly.

Modularity analysis further emphasized the single-node alterations previously identified for the O rules, revealing that these changes prominently affect within-module connectivity, particularly in the DMN and CEN. Disrupted DMN connectivity is a well-established feature of schizophrenia [20], and altered intra-module patterns in the CEN, including reduced connectivity [56], have also been reported. Furthermore, in our analysis, the between-module connectivity of CEN in patients showed (1) increased divergence from the limbic module (more O rules from the limbic module to the CEN) and (2) decreased divergence from the DMN (more S rules from the CEN to the DMN; Figure 9B). At the same time, the basal ganglia emerged as a central hub bridging these major networks, showing increased similarity with the DMN, CEN, and limbic modules (more S rules), while these networks exhibited increased divergence toward the basal ganglia (more O rules; Figure 9C). Overall, these findings support altered DMN–CEN connectivity in schizophrenia, suggesting modulation may involve the basal ganglia and thalamus rather than the salience network as described by the Menon model [24]. Notably, subcortical regions showed minimal within-module changes but exhibited the highest between-module connectivity alterations. The cortico–striato–pallido–thalamo–cortical (CSPTC) circuits have been implicated in schizophrenia pathophysiology [57] and psychedelic-induced brain changes [58], supporting the possible role for subcortical–cortical modulation in large-scale network dysconnectivity. Further refinement of subcortical parcellation could enhance mechanistic insights.

In summary, our findings reveal region-specific alterations in well-known schizophrenia-related networks, including the sensory cortex [59,60] and DMN/CEN [24]. General disinhibition appears to increase connection weights in (1) bottom-up sensory pathways via the ActS rule and (2) within- and between-module associative cortices via ActO/TfO rules, reducing node-state consensus. An altered balance between S and O rules may disrupt dynamic stability and temporal variability, yielding a more random functional network architecture. These alterations align with recent dynamic functional connectivity [32,33,34] and DCM studies [20,29,30,31]. While our results show partial consistency with prior work [20,29,30,54,56], the literature remains mixed, often reporting both hyper- and hypoconnectivity. Notably, our approach estimates activation and deactivation separately, so observed FC increases or decreases may reflect a balance between rules rather than changes in a single type of connectivity. This observation may link neuronal excitation/inhibition (E/I) imbalance to mesoscopic network alterations described by the dysconnectivity hypothesis [61].

### 4.3. Possible Molecular Explanation

Predictive coding is a general model of brain function in which expectations about an event (top-down predictions) are compared with bottom-up sensory inputs to estimate a prediction error. The resulting error is then used to update the prediction of incoming sensory information [62]. Neurobiologically, the precision of a prediction is thought to be encoded in the excitability of superficial pyramidal neurons [52]. NMDA receptors, together with GABAergic interneuron synapses and their modulators (such as dopamine), play a critical role in maintaining this equilibrium. In schizophrenia, abnormal modulation of neuronal excitability is therefore hypothesized to impair the precision of a priori beliefs, giving rise to symptomatology [35]. This framework has already been used to interpret fMRI modulation signals as decreases in self-inhibitory connections in DCM connectivity estimates [29]. In our case, increased TE in the turn-off rules can be interpreted as an index of regional excitability, as enhanced excitability implies that brain regions are more prone to change their state. Under this hypothesis, the strength associated with these rules is expected to be increased in patients. Consistent with this expectation, the TfO rule is the most affected in the patient group. Furthermore, ActO is the second most affected rule in the SCZ group, suggesting that increased excitability preferentially promotes divergence among brain-region states. Taken together, these findings suggest that the instability of brain states in schizophrenia may be driven by increased differentiation dynamics rather than by reduced functional connectivity. Indeed, when acting together, the ActO and TfO rules decrease the probability of similar node states and promote diversification. A potential mechanism involving long-range inhibitory pathways was proposed by Anticevic et al. [63], who described glutamatergic projections (via NMDA receptors) to distant GABAergic interneurons that inhibit their local circuits. Thus, as a hypothetical model, the altered modulation in schizophrenia may reflect enhanced interneuronal modulation across brain regions. Our findings provide strong indications consistent with this interpretation. Furthermore, the modularity analysis suggests a potential role of the basal ganglia in modulating both limbic and DMN modules. The basal ganglia circuitry is a major regulator of cortical activity through dopamine receptors. Therefore, the increased TE from these regions to the cortex is consistent with an index of synaptic modulation, given the hypothesis of altered neuromodulatory processes in schizophrenia. More detailed modeling of subcortical circuits could be particularly useful for estimating TE rules and further testing this hypothesis, as well as integrating pharmacological models to characterize differences in neural interactions. In this context, psychedelic compounds appear especially suitable for probing the neuromodulation of cortical–subcortical circuits [58].

## 5. Conclusions

We introduced a new approach to estimate causal interactions between brain regions and validated it using a database including patients with schizophrenia to explore potential phenotypical characterizations. We could partially confirm the validity of our strategy, but given the dynamic nature of this measure, a different sampling of BOLD signals may affect the reproducibility of our results. In addition, a characterization of altered brain circuits in schizophrenia patients is presented. Several of our findings seem in agreement with previous studies, while some new ideas can be hypothesized and pave the way to future work.

## Figures and Tables

**Table 1:** Interactions between A and B ROIs. The interaction rules between ROIs (left tables) are described as (A_n_), the state of A in a given time step n, and (B_n_), the state of B in the same time step n, producing (B_n+1_), and the state of B in the following time step n + 1. This representation focuses on the possible mechanism of the information transfer from A to B. The formal equations of the conditional probability for each interaction are shown on the right.

Interaction Rules	Conditional Probability $P\left(B_{n+1}|A_{n}^{\left(k\right)};B_{n}^{\left(l\right)}\right)$	Interaction Rules	Conditional Probability $P\left(B_{n+1}|A_{n}^{\left(k\right)};B_{n}^{\left(l\right)}\right)$
A_n_ (0) + B_n_ (0) → B_n+1_ (0)	$P\left(0_{n+1}|0_{n}^{\left(1\right)};0_{n}^{\left(1\right)}\right)$	A_n_ (1) + B_n_ (0) → B_n+1_ (0)	$P\left(0_{n+1}|1_{n}^{\left(1\right)};0_{n}^{\left(1\right)}\right)$
A_n_ (0) + B_n_ (0) → B_n+1_ (1)	$P\left(1_{n+1}|0_{n}^{\left(1\right)};0_{n}^{\left(1\right)}\right)$	A_n_ (1) + B_n_ (0) → B_n+1_ (1)	$P\left(1_{n+1}|1_{n}^{\left(1\right)};0_{n}^{\left(1\right)}\right)$
A_n_ (0) + B_n_ (0) → B_n+1_ (−1)	$P\left({-1}_{n+1}|0_{n}^{\left(1\right)};0_{n}^{\left(1\right)}\right)$	A_n_ (1) + B_n_ (0) → B_n+1_ (−1)	$P\left({-1}_{n+1}|1_{n}^{\left(1\right)};0_{n}^{\left(1\right)}\right)$
A_n_ (1) + B_n_ (1) → B_n+1_ (0)	$P\left(0_{n+1}|1_{n}^{\left(1\right)};1_{n}^{\left(1\right)}\right)$	A_n_ (1) + B_n_ (−1) → B_n+1_ (0)	$P\left(0_{n+1}|1_{n}^{\left(1\right)};{-1}_{n}^{\left(1\right)}\right)$
A_n_ (1) + B_n_ (1) → B_n+1_ (1)	$P\left(1_{n+1}|1_{n}^{\left(1\right)};1_{n}^{\left(1\right)}\right)$	A_n_ (1) + B_n_ (−1) → B_n+1_ (1)	$P\left(1_{n+1}|1_{n}^{\left(1\right)};{-1}_{n}^{\left(1\right)}\right)$
A_n_ (1) + B_n_ (1) → B_n+1_ (−1)	$P\left({-1}_{n+1}|1_{n}^{\left(1\right)};1_{n}^{\left(1\right)}\right)$	A_n_ (1) + B_n_ (−1) → B_n+1_ (−1)	$P\left({-1}_{n+1}|1_{n}^{\left(1\right)};{-1}_{n}^{\left(1\right)}\right)$
A_n_ (−1) + B_n_ (−1) → B_n+1_ (0)	$P\left(0_{n+1}|{-1}_{n}^{\left(1\right)};{-1}_{n}^{\left(1\right)}\right)$	A_n_ (−1) + B_n_ (0) → B_n+1_ (0)	$P\left(0_{n+1}|{-1}_{n}^{\left(1\right)};0_{n}^{\left(1\right)}\right)$
A_n_ (−1) + B_n_ (−1) → B_n+1_ (1)	$P\left(1_{n+1}|{-1}_{n}^{\left(1\right)};-1_{n}^{\left(1\right)}\right)$	A_n_ (−1) + B_n_ (0) → B_n+1_ (1)	$P\left(1_{n+1}|{-1}_{n}^{\left(1\right)};0_{n}^{\left(1\right)}\right)$
A_n_ (−1) + B_n_ (−1) → B_n+1_ (−1)	$P\left({-1}_{n+1}|{-1}_{n}^{\left(1\right)};{-1}_{n}^{\left(1\right)}\right)$	A_n_ (−1) + B_n_ (0) → B_n+1_ (−1)	$P\left({-1}_{n+1}|{-1}_{n}^{\left(1\right)};0_{n}^{\left(1\right)}\right)$
A_n_ (0) + B_n_ (1) → B_n+1_ (0)	$P\left(0_{n+1}|0_{n}^{\left(1\right)};1_{n}^{\left(1\right)}\right)$	A_n_ (−1) + B_n_ (1) → B_n+1_ (0)	$P\left(0_{n+1}|{-1}_{n}^{\left(1\right)};1_{n}^{\left(1\right)}\right)$
A_n_ (0) + B_n_ (1) → B_n+1_ (1)	$P\left(1_{n+1}|0_{n}^{\left(1\right)};1_{n}^{\left(1\right)}\right)$	A_n_ (−1) + B_n_ (1) → B_n+1_ (1)	$P\left(1_{n+1}|{-1}_{n}^{\left(1\right)};1_{n}^{\left(1\right)}\right)$
A_n_ (0) + B_n_ (1) → B_n+1_ (−1)	$P\left({-1}_{n+1}|0_{n}^{\left(1\right)};1_{n}^{\left(1\right)}\right)$	A_n_ (−1) + B_n_ (1) → B_n+1_ (−1)	$P\left({-1}_{n+1}|{-1}_{n}^{\left(1\right)};1_{n}^{\left(1\right)}\right)$
A_n_ (0) + B_n_ (−1) → B_n+1_ (0)	$P\left(0_{n+1}|0_{n}^{\left(1\right)};-1_{n}^{\left(1\right)}\right)$		
A_n_ (0) + B_n_ (−1) → B_n+1_ (1)	$P\left(1_{n+1}|0_{n}^{\left(1\right)};-1_{n}^{\left(1\right)}\right)$		
A_n_ (0) + B_n_ (−1) → B_n+1_ (−1)	$P\left({-1}_{n+1}|0_{n}^{\left(1\right)};-1_{n}^{\left(1\right)}\right)$		

**Table 2:** Total network weight. The mean and standard deviation (S.D.) of the total network weight (W) for SCZ and HS, along with the statistical comparison and the corresponding *p*-value for each network layer, are shown. To note, the SCZ group shows a significant increase in connectivity in each network layer.

	HS Mean ± S.D	SCZ Mean ± S.D	Group Comparison (*p*-Value)
ActS	115.35 ± 13.14	126.86 ± 21.74	4 × 10^−4^
TfS	46.00 ± 2.56	49.81 ± 4.48	1 × 10^−8^
ActO	81.53 ± 7.02	93.27 ± 19.60	6 × 10^−6^
TfO	45.85 ± 3.17	51.81 ± 4.90	7 × 10^−14^

**Table 3:** Reciprocity Index (ρ). Mean and S.D. values of the individual layers and their interactions are reported. Notably, a significant increase in matrix asymmetry in the patient group was observed only for the TfO rule. Asterisks indicate significant differences after Bonferroni correction.

Rules	HS Mean ± S.D.	SCZ Mean ± S.D.	Group Comparison (*p*-Value)
ActS	−0.04 ± 0.02	−0.05 ± 0.03	0.63
TfS	−0.06 ± 0.02	−0.06 ± 0.02	0.23
ActO	−0.04 ± 0.03	−0.04 ± 0.03	0.86
TfO	−0.06 ± 0.02	−0.08 ± 0.02	2 × 10^−5^ *
ActS/TfS	−0.05 ± 0.01	−0.05 ± 0.01	0.27
ActS/ActO	0.04 ± 0.03	0.05 ± 0.04	0.68
ActS/TfO	0.09 ± 0.02	0.10 ± 0.02	0.05
TfS/ActO	0.14 ± 0.02	0.14 ± 0.02	0.04
TfS/TfO	0.05 ± 0.02	0.06 ± 0.02	0.21
ActO/TfO	−0.04 ± 0.02	−0.04 ± 0.01	0.02

Asterisks indicate significant differences after Bonferroni correction.

**Table 4:** In- and out-strength group comparison. Statistical comparisons between groups were performed to assess differences in in- and out-strength across brain regions. All results reported in this study remained statistically significant after applying the Bonferroni correction for multiple comparisons (*p*-value < 0.05, corrected).

In-Strength			Out-Strength			
Brain Regions	HS Mean ± S.D.	SCZ Mean ± S.D.	Brain Regions	HS Mean ± S.D.	SCZ Mean ± S.D.	Rules
SMA Left	0.97 ± 0.26	1.20 ± 0.38	ICC R	0.99 ± 0.23	1.18 ± 0.33	ActS
n.s.			Insula L	0.41 ± 0.10	0.49 ± 0.15	TfS
			SMG pL	0.39 ± 0.10	0.46 ± 0.11	
			Lingual L	0.40 ± 0.10	0.47 ± 0.10	
			Fusiform L	0.39 ± 0.11	0.48± 0.14	
			Planum Polare R	0.42 ± 0.09	0.49 ± 0.13	
			Putamen L	0.48 ± 0.12	0.58 ± 0.14	
Insula R	0.73 ± 0.23	0.96 ± 0.39	SFG L	0.75 ± 0.25	0.97 ± 0.36	ActO
Subcallosal Cortex	0.75 ± 0.29	1.05 ± 0.42	STG p R	0.63 ± 0.17	0.88 ± 0.30	
Frontal Orb L	0.68 ± 0.22	0.90 ± 0.39	MTG a R	0.70 ± 0.23	0.90 ± 0.31	
PHP pL	0.75 ± 0.24	0.96 ± 0.36	MTG p R	0.72 ± 0.19	0.92 ± 0.29	
Frontal Oper L	0.78 ± 0.29	1.03 ± 0.47	MTG t-o L	0.72 ± 0.22	0.90 ± 0.31	
Planum Polare R	0.73 ± 0.25	0.96 ± 0.43	ITG t-o L	0.81 ± 0.22	0.98 ± 0.33	
			Paracingulate L	0.74 ± 0.19	0.91 ± 0.27	
			Precuneous	0.84 ± 0.27	1.03 ± 0.33	
			Fusiform Occ R	0.76 ± 0.22	0.97 ± 0.36	
			HP R	0.70 ± 0.24	0.94 ± 0.35	
			HP L	0.67 ± 0.20	0.94 ± 0.29	
Insula L	0.42 ± 0.13	0.51 ± 0.13	Frontal Pole L	0.43 ± 0.13	0.54 ± 0.12	TfO
Temp Pole R	0.40 ± 0.14	0.49 ± 0.14	MFG L	0.45 ± 0.12	0.54 ± 0.13	
ITG p L	0.43 ± 0.13	0.52 ± 0.16	MTG p R	0.40 ± 0.13	0.49 ± 0.14	
Paracingulate R	0.41 ± 0.13	0.53 ± 0.15	MTG p L	0.41 ± 0.14	0.50 ± 0.13	
Cingulate anterior	0.40 ± 0.13	0.49 ± 0.13	MTG t-o L	0.43 ± 0.09	0.50 ± 0.14	
PHP a L	0.44 ± 0.11	0.55 ± 0.17	ITG t-o L	0.43 ± 0.13	0.53 ± 0.14	
Fusiform Temp aR	0.40 ± 0.14	0.50 ± 0.14	Postcentral Gyrus R	0.38 ± 0.12	0.46 ± 0.12	
Frontal Oper R	0.43 ± 0.15	0.52 ± 0.14	Lat Occ i R	0.40 ± 0.10	0.49 ± 0.13	
Central Oper R	0.41 ± 0.14	0.50 ± 0.13	Lat Occ i L	0.40 ± 0.14	0.51 ± 0.14	
Supracalcarine R	0.38 ± 0.11	0.47 ± 0.14	Cingulate posterior	0.43 ± 0.11	0.52 ± 0.14	
Putamen Right	0.47 ± 0.14	0.57 ± 0.16	Fusiform Temp p L	0.41 ± 0.12	0.50 ± 0.12	
HP R	0.43 ± 0.15	0.54 ± 0.19	Fusi Temp-Occ R	0.43 ± 0.13	0.52 ± 0.14	
			Planum Temp L	0.40 ± 0.12	0.49 ± 0.12	
			Amygdala L	0.42 ± 0.14	0.53 ± 0.13	

**Table 5:** Modules in SCZ and HS. The brain regions divided into sub-networks are shown for both SCZ and HS groups. In the HS group, a different lateralization was found for the FO (right part in module 1 and left part in module 5) and for the aITG (left part in module 7 and right part in module 3). In the SCZ group, a different lateralization was found for the FO (same as in the HS group) and for the pSMG (left part in module 5 and right part in module 1). Minimal anatomical differences were detected between groups: the pSMG appears in module 5 in HS, while in SCZ, only the right part is included in this module; the aITG appears in module 3 in SCZ, while in HS, only the right part is included in this module.

Modules	HS	SCZ
1: Attention and sensory-motor	IC, PreCG; aSTG; pSTG; PostCG; SPL; aSMG; SMA; AC; FO (R); CO; PO; PP; HG; PT	IC; PreCG; aSTG; pSTG; PostCG; SPL; aSMG; pSMG (R); SMA; AC; FO (R); CO; PO; PP; HG; PT
2: Vision	iLOC; ICC; cuneal; LG; TOFusC; OFusG; SCC; OP	iLOC; ICC; Cuneal; LG; TOFusC; OFusG; SCC; OP
3: Limbic	TP; aITG (R); toITG; aPaHC; pPaHC; aTFusC; pTFusC; hippocampus; amygdala	TP; aITG; aPaHC; pPaHC; aTFusC; pTFusC; hippocampus; amygdala
4: BG	Thalamus; caudate; putamen; pallidum; accumbens	Thalamus; caudate; putamen; pallidum; accumbens
5: CEN	IFG tri; IFG oper; toMTG; pSMG; Forb; FO (L)	IFG tri; IFG oper; toMTG; toITG; pSMG (L); Forb; FO (L)
6: DMN	aMTG; pMTG; AG; MedFC; SubCalC; PaCiG; PC; precuneous	aMTG; pMTG; AG; MedFC; SubCalC; PaCiG; PC; precuneous
7: Fronto-temporo-occipital	FP; SFG; MidFG; aITG (L); pITG; sLOC	FP; SFG; MidFG; pITG; sLOC

**Table 6:** Pattern of increased within- and between-modules connectivity in the SCZ group. Only significant (>0.05), after Bonferroni correction, results are shown.

Rules	Within-Modules	Between-Modules
ActS—ActO—TfO—TfS	n.s.	1 → 2; 1 → 4; 1 → 5; 1 → 6; 1 → 7; 2 →4; 2→7; 4 → 2; 5 → 2; 5 → 7; 6 → 2; 6 → 1; 7 → 1; 7 → 2; 7 → 4; 7 → 5
ActS—TfS—TfO	n.s.	1 → 3; 2 → 5; 3 → 5; 7 → 3
ActO—TfO—TfS	n.s.	2 → 1; 4 → 1; 4 → 6; 6 → 5
ActS—ActO—TfS	n.s.	3 → 1; 3 → 6
ActS—ActO—TfO	1; 2	2 → 3; 4 → 3; 5 → 3; 6 → 3; 5 → 4; 4 → 7; 5 → 6; 7 → 6
ActS—TfS	n.s.	3 → 2; 3 → 4; 3 → 7; 6 → 4; 6 → 7
ActO—TfO	4, 5, 6, 7	2 → 6
TfS—TfO	n.s.	4 → 5

## Data Availability

COBRE (Center for Biomedical Research Excellence) dataset of the 1000 Functional Connectomes Classic collection: http://fcon_1000.projects.nitrc.org/indi/retro/cobre.html (accessed on 14 February 2018).

## Supplementary Materials

The following supporting information can be downloaded at: https://www.mdpi.com/article/10.3390/e28010083/s1. S1 brain atlas; S2 statistical characterization of interaction rules; S3 network density analysis; S4 motif analysis and S5 centrality analysis;

## Abbreviations

The following abbreviations are used in this manuscript:

ActS	activation same
ActO	activation opposite
TfS	turn-off same
TfO	turn-off opposite
DCM	dynamic causal modeling
rsFC	resting state functional connectivity
dFC	dynamic functional connectivity
EC	effective connectivity
TE	transfer entropy
NE	noradrenalina
5-HT	5-hydroxytryptamine
DA	dopamine
DMN	default mode network
SN	salience network
CEN	central executive network

## References

[1] Friston K.J. (2011). Functional and effective connectivity: A review. Brain Connect..

[2] Stephan K.E., Harrison L.M., Penny W.D., Friston K.J. (2004). Biophysical models of fMRI responses. Curr. Opin. Neurobiol..

[3] Razi A., Kahan J., Rees G., Friston K.J. (2015). Construct validation of a DCM for resting state fMRI. Neuroimage.

[4] Seth A.K., Barrett A.B., Barnett L. (2015). Granger causality analysis in neuroscience and neuroimaging. J. Neurosci..

[5] Dsouza A.M., Abidin A.Z., Leistritz L., Wismüller A. (2017). Exploring connectivity with large-scale Granger causality on resting-state functional fMRI. J. Neurosci. Methods.

[6] Vicente R., Wibral M., Lindner M., Pipa G. (2010). Transfer entropy—A model-free measure of effective connectivity for the neurosciences. J. Comput. Neurosci..

[7] Lizier J.T., Prokopenko M., Zomaya A.Y. (2008). Local information transfer as a spatiotemporal filter for complex systems. Phys. Rev. E.

[8] Schwab S., Harbord R., Zerbi V., Elliott L., Afyouni S., Smith J.Q., Woolrich M.W., Smith S.M., Nichols T.E. (2018). Directional functional connectivity using dynamic graphical models. Neuroimage.

[9] Ramsey J.D., Hanson S., Hanson C., Halchenko Y., Poldrack R., Glymour C. (2009). Six problems for causal inference from fMRI. Neuroimage.

[10] Parente F., Colosimo A. (2021). Modelling a multiplex brain network by local transfer entropy. Sci. Rep..

[11] Tagliazucchi E., Balenzuela P., Fraiman D., Chialvo D.R. (2012). Criticality in large-scale brain fMRI dynamics unveiled by a novel point process analysis. Front. Physiol..

[12] Petridou N., Gaudes C.C., Dryden I.L., Francis S.T., Gowland P.A. (2013). Periods of rest in fMRI contain individual spontaneous events which are related to slowly fluctuating spontaneous activity. Hum. Brain Mapp..

[13] Drew P.J. (2019). Vascular and neural basis of the BOLD signal. Curr. Opin. Neurobiol..

[14] Boccaletti S., Bianconi G., Criado R., del Genio C.I., Gómez-Gardeñes J., Romance M., Sendiña-Nadal I., Wang Z., Zanin M. (2014). The structure and dynamics of multilayer networks. Phys. Rep..

[15] Battiston F., Nicosia V., Latora V. (2014). Structural measures for multiplex networks. Phys. Rev. E Stat. Nonlinear Soft Matter Phys..

[16] Parente F., Colosimo A. (2020). Functional connections between and within brain subnetworks under resting-state. Sci. Rep..

[17] Stephan K.E., Friston K.J., Frith C.D. (2009). Dysconnection in schizophrenia: From abnormal synaptic plasticity to failures of self-monitoring. Schizophr. Bull..

[18] Jiang T., Zhou Y., Liu B., Liu Y., Song M. (2013). Brainnetome-wide association studies in schizophrenia: The advances and future. Neurosci. Biobehav. Rev..

[19] Friston K., Brown H.R., Siemerkus J., Stephan K.E. (2016). The dysconnection hypothesis. Schizophr. Res..

[20] Bastos-Leite A.J., Ridgway G.R., Silveira C., Norton A., Reis S., Friston K.J. (2015). Dysconnectivity within the default mode in first-episode schizophrenia: A stochastic dynamic causal modeling study with functional magnetic resonance imaging. Schizophr. Bull..

[21] Schiwy L.C., Forlim C.G., Fischer D.J., Kühn S., Becker M., Gallinat J. (2022). Aberrant functional connectivity within the salience network is related to cognitive deficits and disorganization in psychosis. Schizophr. Res..

[22] Chahine G., Richter A., Wolter S., Goya-Maldonado R., Gruber O. (2017). Disruptions in the left frontoparietal network underlie resting-state endophenotypic markers in schizophrenia. Hum. Brain Mapp..

[23] Zhuo C., Wang C., Wang L., Guo X., Xu Q., Liu Y., Zhu J. (2018). Altered resting-state functional connectivity of the cerebellum in schizophrenia. Brain Imaging Behav..

[24] Menon V. (2011). Large-scale brain networks and psychopathology: A unifying triple network model. Trends Cogn. Sci..

[25] Supekar K., Cai W., Krishnadas R., Palaniyappan L., Menon V. (2019). Dysregulated brain dynamics in a triple-network saliency model of schizophrenia and its relation to psychosis. Biol. Psychiatry.

[26] Liao X., Vasilakos A.V., He Y. (2017). Small-world human brain networks: Perspectives and challenges. Neurosci. Biobehav. Rev..

[27] Bassett D.S., Bullmore E., Verchinski B.A., Mattay V.S., Weinberger D.R., Meyer-Lindenberg A. (2008). Hierarchical organization of human cortical networks in health and schizophrenia. J. Neurosci..

[28] Lynall M.E., Bassett D.S., Kerwin R., McKenna P.J., Kitzbichler M., Müller U., Bullmore E. (2010). Functional connectivity and brain networks in schizophrenia. J. Neurosci..

[29] Zarghami T.S., Zeidman P., Razi A., Bahrami F., Hossein-Zadeh G.A. (2023). Dysconnection and cognition in schizophrenia: A spectral dynamic causal modeling study. Hum. Brain Mapp..

[30] Rolls E.T., Cheng W., Gilson M., Gong W., Deco G., Lo C.-Y.Z., Yang A.C., Tsai S.-J., Liu M.-E., Lin C.-P. (2020). Beyond the disconnectivity hypothesis of schizophrenia. Cereb. Cortex.

[31] Rolls E.T. (2021). Attractor cortical neurodynamics, schizophrenia, and depression. Transl. Psychiatry.

[32] Ramirez-Mahaluf J.P., Tepper Á., Alliende L.M., Mena C., Castañeda C.P., Iruretagoyena B., Nachar R., Reyes-Madrigal F., León-Ortiz P., Mora-Durán R. (2023). Dysconnectivity in schizophrenia revisited: Abnormal temporal organization of dynamic functional connectivity in patients with a first episode of psychosis. Schizophr. Bull..

[33] Weber S., Johnsen E., Kroken R.A., Løberg E.M., Kandilarova S., Stoyanov D., Kompus K., Hugdahl K. (2020). Dynamic functional connectivity patterns in schizophrenia and the relationship with hallucinations. Front. Psychiatry.

[34] Damaraju E., Allen E.A., Belger A., Ford J., McEwen S., Mathalon D., Mueller B., Pearlson G., Potkin S., Preda A. (2014). Dynamic functional connectivity analysis reveals transient states of dysconnectivity in schizophrenia. Neuroimage Clin..

[35] Adams R.A., Stephan K.E., Brown H.R., Frith C.D., Friston K.J. (2013). The computational anatomy of psychosis. Front. Psychiatry.

[36] Rashid B., Damaraju E., Pearlson G.D., Calhoun V.D. (2014). Dynamic connectivity states estimated from resting fMRI Identify differences among Schizophrenia, bipolar disorder, and healthy control subjects. Front. Hum. Neurosci..

[37] Du Y., Pearlson G.D., Lin D., Sui J., Chen J., Salman M., Tamminga C.A., Ivleva E.I., Sweeney J.A., Keshavan M.S. (2017). Identifying dynamic functional connectivity biomarkers using GIG-ICA: Application to schizophrenia, schizoaffective disorder, and psychotic bipolar disorder. Hum. Brain Mapp..

[38] Power J.D., Mitra A., Laumann T.O., Snyder A.Z., Schlaggar B.L., Petersen S.E. (2014). Methods to detect, characterize, and remove motion artifact in resting state fMRI. Neuroimage.

[39] Behzadi Y., Restom K., Liau J., Liu T.T. (2007). A component based noise correction method (CompCor) for BOLD and perfusion based fMRI. Neuroimage.

[40] Desikan R.S., Ségonne F., Fischl B., Quinn B.T., Dickerson B.C., Blacker D., Buckner R.L., Dale A.M., Maguire R.P., Hyman B.T. (2006). An automated labeling system for subdividing the human cerebral cortex on MRI scans into gyral based regions of interest. Neuroimage.

[41] Rokach L., Maimon O., Maimon O., Rokach L. (2005). Clustering methods. Data Mining and Knowledge Discovery Handbook.

[42] Squartini T., Picciolo F., Ruzzenenti F., Garlaschelli D. (2013). Reciprocity of weighted networks. Sci. Rep..

[43] Gemmetto V., Squartini T., Picciolo F., Ruzzenenti F., Garlaschelli D. (2016). Multiplexity and multireciprocity in directional multiplexes. Phys. Rev. E.

[44] Dimitrova T., Petrovski K., Kocarev L. (2020). Graphlets in multiplex networks. Sci. Rep..

[45] Takaguchi T., Yoshida Y. (2016). Cycle and flow trusses in directional networks. R. Soc. Open Sci..

[46] Onnela J.P., Saramäki J., Kertész J., Kaski K. (2005). Intensity and coherence of motifs in weighted complex networks. Phys. Rev. E.

[47] Borgatti S.P., Everett M.G. (2006). A graph-theoretic perspective on centrality. Soc. Netw..

[48] Newman M.E.J. (2008). Community structure in directional networks. Phys. Rev. Lett..

[49] Rubinov M., Sporns O. (2009). Complex network measures of brain connectivity: Uses and interpretations. Neuroimage.

[50] Rubinov M., Sporns O. (2011). Weight-conserving characterization of complex functional brain networks. Neuroimage.

[51] Rolls E.T., Cheng W., Feng J. (2021). Brain dynamics: Synchronous peaks, functional connectivity, and its temporal variability. Hum. Brain Mapp..

[52] Bastos A.M., Usrey W.M., Adams R.A., Mangun G.R., Fries P., Friston K.J. (2012). Canonical microcircuits for predictive coding. Neuron.

[53] Picó-Pérez M., Vieira R., Fernández-Rodríguez M., De Barros M.A.P., Radua J., Morgado P. (2022). Multimodal meta-analysis of structural gray matter, neurocognitive and social cognitive fMRI findings in schizophrenia patients. Psychol. Med..

[54] Ishida T., Nakamura Y., Tanaka S.C., Mitsuyama Y., Yokoyama S., Shinzato H., Itai E., Okada G., Kobayashi Y., Kawashima T. (2023). Aberrant large-scale network interactions across psychiatric disorders revealed by large-sample multi-site resting-state functional magnetic resonance imaging datasets. Schizophr. Bull..

[55] Parente F., Frascarelli M., Mirigliani A., Di Fabio F., Biondi M., Colosimo A. (2018). Negative functional brain networks. Brain Imaging Behav..

[56] Rong B., Huang H., Gao G., Sun L., Zhou Y., Xiao L., Wang H., Wang G. (2023). Widespread intra- and inter-network dysconnectivity among large-scale resting state networks in schizophrenia. J. Clin. Med..

[57] Avram M., Brandl F., Bäuml J., Sorg C. (2018). Cortico-thalamic hypo- and hyperconnectivity extend consistently to basal ganglia in schizophrenia. Neuropsychopharmacology.

[58] Avram M., Rogg H., Korda A., Andreou C., Müller F., Borgwardt S. (2021). Bridging the gap? Altered thalamocortical connectivity in psychotic and psychedelic states. Front. Psychiatry.

[59] Carment L., Dupin L., Guedj L., Térémetz M., Krebs M.-O., Cuenca M., A Maier M., Amado I., Lindberg P.G. (2019). Impaired attentional modulation of sensorimotor control and cortical excitability in schizophrenia. Brain.

[60] Braff D.L., Light G.A. (2005). The use of neurophysiological endophenotypes to understand the genetic basis of schizophrenia. Dialogues Clin. Neurosci..

[61] Anticevic A., Lisman J. (2017). How can global alteration of excitation/inhibition balance lead to the local dysfunctions that underlie schizophrenia?. Biol. Psychiatry.

[62] Friston K. (2008). Hierarchical models in the brain. PLoS Comput. Biol..

[63] Anticevic A., Gancsos M., Murray J.D., Repovs G., Driesen N.R., Ennis D.J., Niciu M.J., Morgan P.T., Surti T.S., Bloch M.H. (2012). NMDA receptor function in large-scale anticorrelated neural systems with implications for cognition and schizophrenia. Proc. Natl. Acad. Sci. USA.

