# Supplementary material for: Understanding Schizophrenia Pathophysiology via fMRI-Based Information Theory and Multiplex Network Analysis
Source: Entropy (Basel). 2026 Jan 10;28(1):83. doi: 10.3390/e28010083 (PMC12839680; doi:10.3390/e28010083)
Supplement: Supplementary file 1 [file entropy-28-00083-s001.zip › entropy-3997542-supplementary.pdf]

Understanding schizophrenia pathophysiology via fMRI-based information theory and multiplex network analysis

Fabrizio Parente[1]
1. Affiliation 1: Department of Physiology and Pharmacology, Sapienza University of Rome, P.le A. Moro 5, 00185 Rome, Italy

(SUPPLEMENTARY MATERIALS)

S1 – Information about brain atlas

S2 – Statistical characterization of interaction rules

S3 – Network Density Analysis

S4 – Motif analysis

S5 – Centrality Analysis

S1. Information about brain atlas

Table S1. Brain regions. The whole set of ROIs by the Harvard-Oxford atlas [1]. The first 105 ROIs were used for the analysis (i.e., excluding brainstem and cerebellum).

1	'harvard-oxford.FP r (Frontal Pole Right)'
2	'harvard-oxford.FP l (Frontal Pole Left)'
3	'harvard-oxford.IC r (Insular Cortex Right)'
4	'harvard-oxford.IC l (Insular Cortex Left)'
5	'harvard-oxford.SFG r (Superior Frontal Gyrus Right)'
6	'harvard-oxford.SFG l (Superior Frontal Gyrus Left)'
7	'harvard-oxford.MidFG r (Middle Frontal Gyrus Right)'
8	'harvard-oxford.MidFG l (Middle Frontal Gyrus Left)'
9	'harvard-oxford.IFG tri r (Inferior Frontal Gyrus, pars triangularis Right)'
10	'harvard-oxford.IFG tri l (Inferior Frontal Gyrus, pars triangularis Left)'
11	'harvard-oxford.IFG oper r (Inferior Frontal Gyrus, pars opercularis Right)'
12	'harvard-oxford.IFG oper l (Inferior Frontal Gyrus, pars opercularis Left)'
13	'harvard-oxford.PreCG r (Precentral Gyrus Right)'
14	'harvard-oxford.PreCG l (Precentral Gyrus Left)'
15	'harvard-oxford.TP r (Temporal Pole Right)'
16	'harvard-oxford.TP l (Temporal Pole Left)'
17	'harvard-oxford.aSTG r (Superior Temporal Gyrus, anterior division Right)'
18	'harvard-oxford.aSTG l (Superior Temporal Gyrus, anterior division Left)'
19	'harvard-oxford.pSTG r (Superior Temporal Gyrus, posterior division Right)'
20	'harvard-oxford.pSTG l (Superior Temporal Gyrus, posterior division Left)'
21	'harvard-oxford.aMTG r (Middle Temporal Gyrus, anterior division Right)'
22	'harvard-oxford.aMTG l (Middle Temporal Gyrus, anterior division Left)'
23	'harvard-oxford.pMTG r (Middle Temporal Gyrus, posterior division Right)'
24	'harvard-oxford.pMTG l (Middle Temporal Gyrus, posterior division Left)'
25	'harvard-oxford.toMTG r (Middle Temporal Gyrus, temporooccipital part Right)'
26	'harvard-oxford.toMTG l (Middle Temporal Gyrus, temporooccipital part Left)'
27	'harvard-oxford.aITG r (Inferior Temporal Gyrus, anterior division Right)'
28	'harvard-oxford.aITG l (Inferior Temporal Gyrus, anterior division Left)'
29	'harvard-oxford.pITG r (Inferior Temporal Gyrus, posterior division Right)'
30	'harvard-oxford.pITG l (Inferior Temporal Gyrus, posterior division Left)'
31	'harvard-oxford.toITG r (Inferior Temporal Gyrus, temporooccipital part Right)'
32	'harvard-oxford.toITG l (Inferior Temporal Gyrus, temporooccipital part Left)'
33	'harvard-oxford.PostCG r (Postcentral Gyrus Right)'
34	'harvard-oxford.PostCG l (Postcentral Gyrus Left)'
35	'harvard-oxford.SPL r (Superior Parietal Lobule Right)'
36	'harvard-oxford.SPL l (Superior Parietal Lobule Left)'
37	'harvard-oxford.aSMG r (Supramarginal Gyrus, anterior division Right)'
38	'harvard-oxford.aSMG l (Supramarginal Gyrus, anterior division Left)'
39	'harvard-oxford.pSMG r (Supramarginal Gyrus, posterior division Right)'
40	'harvard-oxford.pSMG l (Supramarginal Gyrus, posterior division Left)'
41	'harvard-oxford.AG r (Angular Gyrus Right)'
42	'harvard-oxford.AG l (Angular Gyrus Left)'
43	'harvard-oxford.sLOC r (Lateral Occipital Cortex, superior division Right)'
44	'harvard-oxford.sLOC l (Lateral Occipital Cortex, superior division Left)'
45	'harvard-oxford.iLOC r (Lateral Occipital Cortex, inferior division Right)'
46	'harvard-oxford.iLOC l (Lateral Occipital Cortex, inferior division Left)'
47	'harvard-oxford.ICC r (Intracalcarine Cortex Right)'
48	'harvard-oxford.ICC l (Intracalcarine Cortex Left)'
49	'harvard-oxford.MedFC (Frontal Medial Cortex)'
50	'harvard-oxford.SMA r (Juxtapositional Lobule Cortex -formerly Supplementary I
51	'harvard-oxford.SMA L (Juxtapositional Lobule Cortex -formerly Supplementary I
52	'harvard-oxford.SubCalC (Subcallosal Cortex)'
53	'harvard-oxford.PaCiG r (Paracingulate Gyrus Right)'
54	'harvard-oxford.PaCiG l (Paracingulate Gyrus Left)'
55	'harvard-oxford.AC (Cingulate Gyrus, anterior division)'
56	'harvard-oxford.PC (Cingulate Gyrus, posterior division)'
57	'harvard-oxford.Precuneous (Precuneous Cortex)'
58	'harvard-oxford.Cuneal r (Cuneal Cortex Right)'
59	'harvard-oxford.Cuneal l (Cuneal Cortex Left)'
60	'harvard-oxford.FOrb r (Frontal Orbital Cortex Right)'
61	'harvard-oxford.FOrb l (Frontal Orbital Cortex Left)'
62	'harvard-oxford.aPaHC r (Parahippocampal Gyrus, anterior division Right)'
63	'harvard-oxford.aPaHC l (Parahippocampal Gyrus, anterior division Left)'
64	'harvard-oxford.pPaHC r (Parahippocampal Gyrus, posterior division Right)'
65	'harvard-oxford.pPaHC l (Parahippocampal Gyrus, posterior division Left)'
66	'harvard-oxford.LG r (Lingual Gyrus Right)'
67	'harvard-oxford.LG l (Lingual Gyrus Left)'
68	'harvard-oxford.aTFusC r (Temporal Fusiform Cortex, anterior division Right)'
69	'harvard-oxford.aTFusC l (Temporal Fusiform Cortex, anterior division Left)'
70	'harvard-oxford.pTFusC r (Temporal Fusiform Cortex, posterior division Right)'
71	'harvard-oxford.pTFusC l (Temporal Fusiform Cortex, posterior division Left)'
72	'harvard-oxford.TOFusC r (Temporal Occipital Fusiform Cortex Right)'
73	'harvard-oxford.TOFusC l (Temporal Occipital Fusiform Cortex Left)'
74	'harvard-oxford.OFusG r (Occipital Fusiform Gyrus Right)'
75	'harvard-oxford.OFusG l (Occipital Fusiform Gyrus Left)'
76	'harvard-oxford.FO r (Frontal Operculum Cortex Right)'
77	'harvard-oxford.FO l (Frontal Operculum Cortex Left)'
78	'harvard-oxford.CO r (Central Opercular Cortex Right)'
79	'harvard-oxford.CO l (Central Opercular Cortex Left)'
80	'harvard-oxford.PO r (Parietal Operculum Cortex Right)'
81	'harvard-oxford.PO l (Parietal Operculum Cortex Left)'
82	'harvard-oxford.PP r (Planum Polare Right)'
83	'harvard-oxford.PP l (Planum Polare Left)'
84	'harvard-oxford.HG r (Heschl''s Gyrus Right)'
85	'harvard-oxford.HG l (Heschl''s Gyrus Left)'
86	'harvard-oxford.PT r (Planum Temporale Right)'
87	'harvard-oxford.PT l (Planum Temporale Left)'
88	'harvard-oxford.SCC r (Supracalcarine Cortex Right)'
89	'harvard-oxford.SCC l (Supracalcarine Cortex Left)'
90	'harvard-oxford.OP r (Occipital Pole Right)'
91	'harvard-oxford.OP l (Occipital Pole Left)'
92	'harvard-oxford.Thalamus r'
93	'harvard-oxford.Thalamus l'
94	'harvard-oxford.Caudate r'
95	'harvard-oxford.Caudate l'
96	'harvard-oxford.Putamen r'
97	'harvard-oxford.Putamen l'
98	'harvard-oxford.Pallidum r'
99	'harvard-oxford.Pallidum l'
100	'harvard-oxford.Hippocampus r'
101	'harvard-oxford.Hippocampus l'
102	'harvard-oxford.Amygdala r'
103	'harvard-oxford.Amygdala l'
104	'harvard-oxford.Accumbens r'
105	'harvard-oxford.Accumbens l'
106	'harvard-oxford.Brain-Stem'
107	'harvard-oxford.Cereb1 l (Cerebelum Crus1 Left)'
108	'harvard-oxford.Cereb1 r (Cerebelum Crus1 Right)'
109	'harvard-oxford.Cereb2 l (Cerebelum Crus2 Left)'
110	'harvard-oxford.Cereb2 r (Cerebelum Crus2 Right)'
111	'harvard-oxford.Cereb3 l (Cerebelum 3 Left)'
112	'harvard-oxford.Cereb3 r (Cerebelum 3 Right)'
113	'harvard-oxford.Cereb45 l (Cerebelum 4 5 Left)'
114	'harvard-oxford.Cereb45 r (Cerebelum 4 5 Right)'
115	'harvard-oxford.Cereb6 l (Cerebelum 6 Left)'
116	'harvard-oxford.Cereb6 r (Cerebelum 6 Right)'
117	'harvard-oxford.Cereb7 l (Cerebelum 7b Left)'
118	'harvard-oxford.Cereb7 r (Cerebelum 7b Right)'
119	'harvard-oxford.Cereb8 l (Cerebelum 8 Left)'
120	'harvard-oxford.Cereb8 r (Cerebelum 8 Right)'
121	'harvard-oxford.Cereb9 l (Cerebelum 9 Left)'
122	'harvard-oxford.Cereb9 r (Cerebelum 9 Right)'
123	'harvard-oxford.Cereb10 l (Cerebelum 10 Left)'
124	'harvard-oxford.Cereb10 r (Cerebelum 10 Right)'
125	'harvard-oxford.Ver12 (Vermis 1 2)'
126	'harvard-oxford.Ver3 (Vermis 3)'
127	'harvard-oxford.Ver45 (Vermis 4 5)'
128	'harvard-oxford.Ver6 (Vermis 6)'
129	'harvard-oxford.Ver7 (Vermis 7)'
130	'harvard-oxford.Ver8 (Vermis 8)'
131	'harvard-oxford.Ver9 (Vermis 9)'
132	'harvard-oxford.Ver10 (Vermis 10)'

S2. Statistical characterization of interaction rules

In panels A of Figures S1 and S2, the set of rules showing an increasing trend of TE as a function of the signal threshold is displayed for the control and SCZ groups, respectively. The analysis indicates that twelve types of interactions exhibit the same trend in MSE values (panel B), reflecting an increased amount of information compared to the random model as a function of the threshold. The remaining rules (panels B and C in both Figures S1 and S2) show a decreasing or mixed trend, suggesting potential sources of noise. ANOVA performed on the previously characterized brain interactions at the higher signal threshold (1 S.D.) confirmed a significant increase in local TE for only eight of the twelve rules mentioned above (Figure S3, left panel), with similar results obtained for both groups.

The correlation among these significant rules was investigated using both correlation analysis and a hierarchical clustering method. In the middle panel of Figure S3, the results of the correlation analysis are shown. A strong relationship appears between combinations of states that are hypothesized to share a similar underlying mechanism: 1) A_n (1) + B_n (0) $\rightarrow$ B_{n+1} (1) and A_n (-1) + B_n (0) $\rightarrow$ B_{n+1} (-1); 2) A_n (1) + B_n (-1) $\rightarrow$ B_{n+1} (0) and A_n (-1) + B_n (1) $\rightarrow$ B_{n+1} (0); 3) A_n (1) + B_n (0) $\rightarrow$ B_{n+1} (-1) and A_n (-1) + B_n (0) $\rightarrow$ B_{n+1} (1); 4) A_n (1) + B_n (1) $\rightarrow$ B_{n+1} (0) and A_n (-1) + B_n (-1) $\rightarrow$ B_{n+1} (0). According to our previous work [2], these four interactions were called, respectively: 1) Activation Same (ActS); 2) Turn off Same (TfS); 3) Activation Opposite (ActO); and 4) Turn off Opposite (TfO). In the same analysis (Figure S3, middle), another positive relationship can be observed between 1) ActS and TfS, and 2) ActO and TfO. These additional sets are referred to, respectively, as Same (S) and Opposite (O). However, the TfS rule appears correlated with the O rules, in contrast to our previous work [2]. The hierarchical clustering analysis (Figure S3, right) confirms the correlation analysis and identifies a first level of clustering corresponding to the combination of the four rules mentioned above (ActS, TfS, ActO, TfO). A second level groups the O rules together (ActO and TfO). At the third and fourth levels, TfS and ActS are sequentially added to the previous O cluster, respectively.

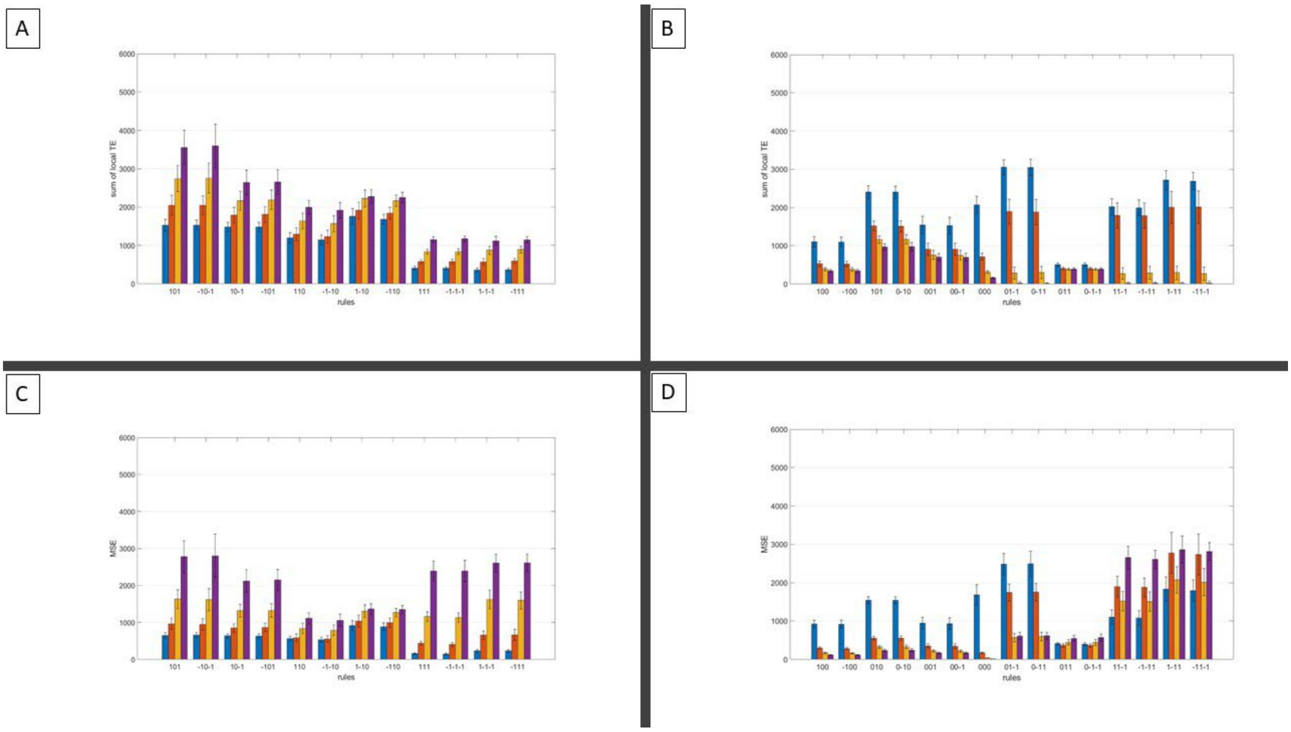

Figure S1. Sums of local TE and MSE values for the interaction rules in the control group. Panels A and C show the set of rules exhibiting an increasing trend in TE and MSE, respectively, as a function of the signal threshold. Panels B and D show the set of rules with a decreasing or mixed trend, suggesting potential sources of noise.

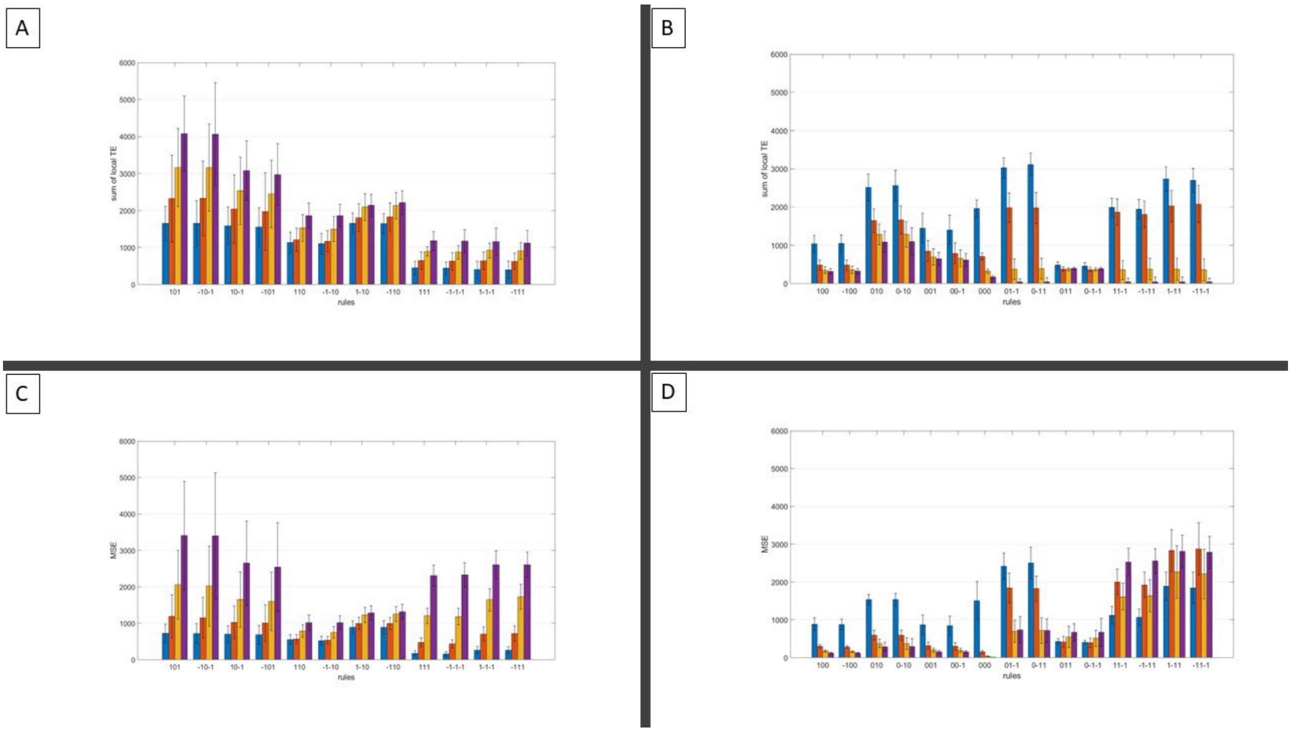

Figure S2. Sums of local TE and MSE values for the interaction rules in the SCZ group. Panels A and C show the set of rules exhibiting an increasing trend in TE and MSE, respectively, as a function of the signal threshold. Panels B and D show the set of rules with a decreasing or mixed trend, suggesting potential sources of noise.

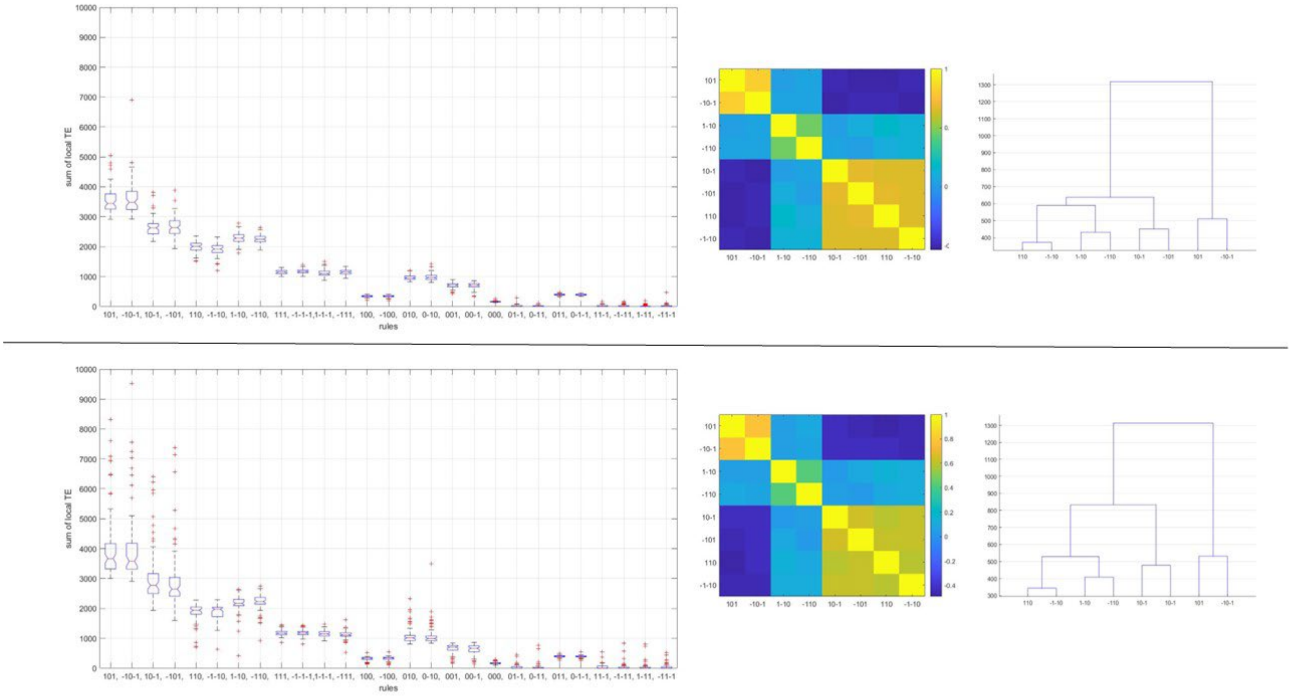

Figure S3. **ANOVA and clustering analysis**. Upper and bottom panel refers to controls and SCZ group, respectively. Left part: ANOVA analysis; right part: hierarchical clustering analysis.

S3. Network Density Analysis

The network density for each rule was calculated for both groups. This measure counts the number of network links, normalized by the maximum possible number of edges. Figures S4 and S5 show the group distributions for the total network weight and the network density, respectively. The SCZ group exhibits

significantly increased density values for all interaction rules considered. See Table S2 for a quantitative description of the measures and statistical results.

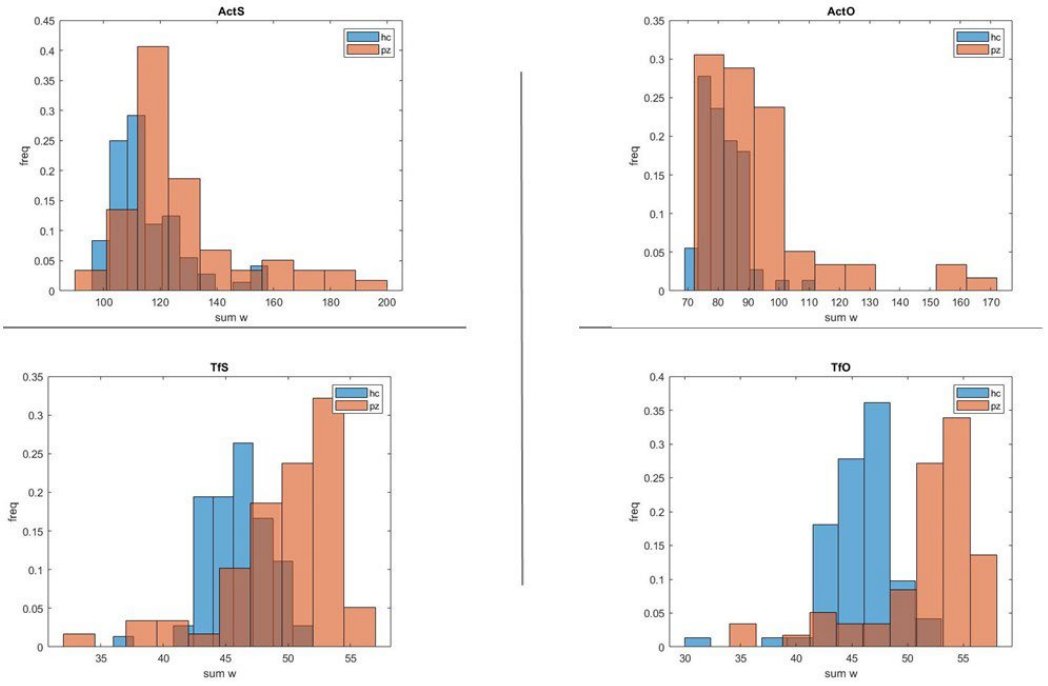

Figure S4. Total network weight distribution. Blue = healthy controls; Red= patients. Note that in the patient group, weight density appears increased in every rule compared to the controls.

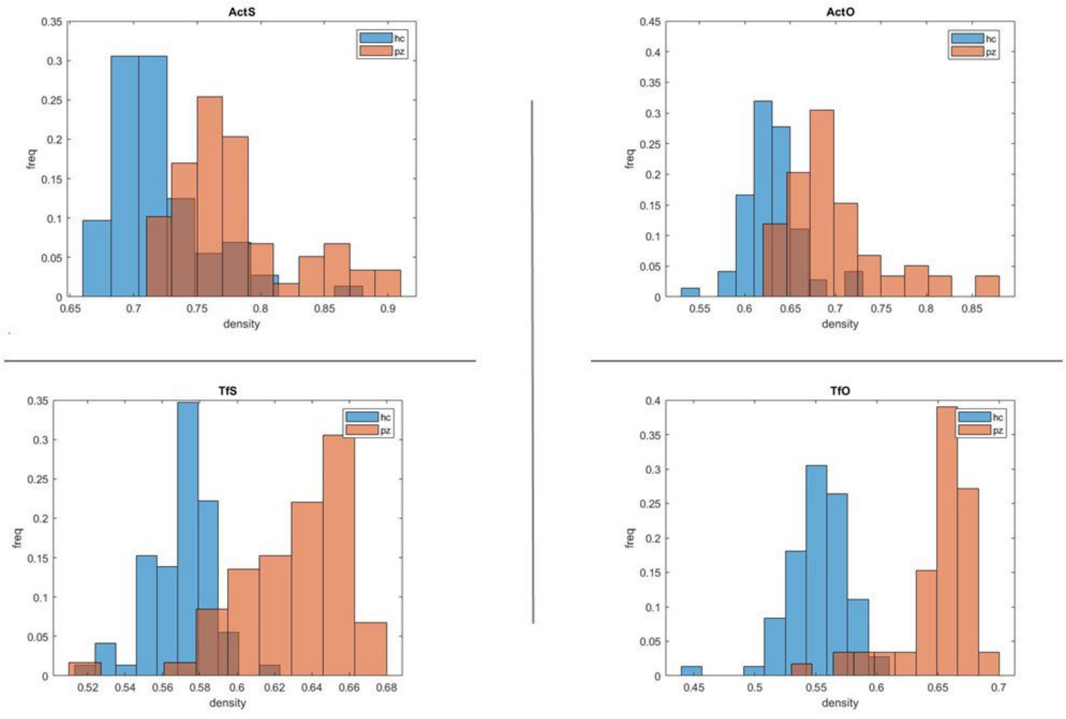

Figure S5. Network density distribution. Blue = healthy controls; Red= patients. Note that in the patient group, weight density appears increased in every rule compared to the controls.

Table S2. Network density analysis. Mean and S.D. values of patients (SCZ) and controls (HC), and statistical comparison for each network layer.

	HC	SCA	p-value
**ActS**	0.72 ± 0.04	0.78 ± 0.05	$2*10^{-13}$
**TfS**	0.57 ± 0.02	0.63 ± 0.03	$4*10^{-29}$
**ActO**	0.63 ± 0.03	0.70 ± 0.05	$4*10^{-16}$
**TfO**	0.55 ± 0.02	0.65 ± 0.03	$5*10^{-43}$

S4. Subgraph analysis

The statistical comparison of the 0th-order motifs (see Table S3) shows a general trend of increased values in the patient group, except for the open triangles of the ActS rule, while the corresponding subgraphs in the ActO rule are non-significant. A similar pattern is observed for the 1st- and 2nd-order motif analyses (Tables S4 and S5), in which the patient group shows generally increased values for all motifs, except for the 1st-order open triangles within the ActS and ActO rules, as well as the ActS and TfO rules.

Table S3. **0-order motif analysis, statistical comparison.** (*) significant, (**) significant Bonferroni.

0-order			
**motif**	**HC**	**SCZ**	**p-value**
Cycle ActS**	1477.1 ± 349	1953.4 ± 660.8	$4.3*10^{-9}$
Flux ActS**	4932.5 ± 1166.5	6360.1 ± 2088.4	$9.9*10^{-8}$
Chain ActS**	1981.5 ± 171.6	1792.3 ± 132.7	$2.9*10^{-11}$
Source ActS**	1775.6 ± 133.5	1597.3 ± 115.1	$1.8*10^{-14}$
Sink ActS **	1808.6 ± 118.9	1633.8 ± 126.7	$2.2*10^{-13}$
Cycle TftS**	397.9 ± 46.1	542.2 ± 94.3	$3.4*10^{-15}$
Flux TfS**	1271.7 ± 126.8	1702 ± 268.3	$3.7*10^{-16}$
Chain TfS**	985.7 ± 46.4	1008.4 ± 67.7	0.0005
Source TfS**	1004.7 ± 45.8	1037.5 ± 64.5	$6.8*10^{-6}$
Sink TfS*	1034 ± 58.3	1055.3 ± 70.3	0.002
Cycle ActO**	800.4 ± 144.8	1174.3 ± 530.7	$6.3*10\text{-}^{12}$
Flux ActO**	2491 ± 474.8	3645.3 ± 1658.3	$3.9*10^{-12}$
Chain ActO	1515.6 ± 96.8	1520.1 ± 111.7	0.87
Source ActO	1639.1 ± 121.2	1604.5 ± 136.4	0.07
Sink ActO	1661.3 ± 99.1	1654.7 ± 122.2	0.57
Cycle TfO**	364.8 ± 57	585.4 ± 100.4	$6.9*10^{-18}$
Flux TfO**	1169.3 ± 155.6	1864 ± 295.5	$5.2*10^{-19}$
Chain TfO**	967.9 ± 64.7	1029.4 ± 74.1	$2.9*10^{-9}$
Source TfO*	1022.9 ± 64.8	1035 ± 62.5	0.03
Sink TfO	1046.3 ± 70.9	1052.8 ± 72	0.11

Table S4. **1-order motif analysis, statistical comparison.** (*) significant, (**) significant Bonferroni.

motif	ctrl	patients	p-value
Cycle ActS ActS TfS**	605.8 ± 41.1	809 ± 77	$1.8*10^{-21}$
Cycle TfS ActS ActS**	934.4 ± 115.6	1231.6 ± 209.6	$5.1*10^{-17}$
Flux ActS TfS/TfS**	1993.7 ± 143.5	2592.4 ± 228.1	$1.6*10^{-21}$
Flux TfS ActS/ActS**	2995.8 ± 395.3	3875.7 ± 666	$4.2*10^{-15}$
Flux TfS ActS/TfS**	1955 ± 132.8	2568.5 ± 235.1	$6.91*10^{-22}$
Flux ActS TfS/ ActS**	2978.1 ± 389.9	3855.6 ± 656.7	$1.7*10^{-15}$
Flux TfS TfS/ActS**	1888 ± 129.3	2478.5 ± 240.7	$6.4*10^{-21}$
Flux ActS ActS/TfS**	3007.4 ± 394.3	3884.5 ± 654.3	$2.6*10^{-15}$
Chain ActS/TfS**	2324.2 ± 123.9	2393 ± 106.7	0.0001
Chain TfS/ActS**	2309.7 ± 104.3	2383.3 ± 108.7	$5.2*10^{-5}$
Source ActS/TfS**	4544.1 ± 235.8	4680.4 ± 198.9	$7.3*10^{-5}$
Sink ActS/TfS**	4571.1 ± 228.5	4707.7 ± 208.9	$6.9*10^{-5}$
Cycle ActS ActS ActO**	987.5 + 170.5	1399.5 ± 547.9	$1*10^{-14}$
Cycle ActO ActS ActS**	1263.1 + 237.8	1697.2 ± 592.9	$9*10^{-12}$
Flux ActS ActO/ActO**	3172.5 + 530.3	4425.1 ± 1687	$5.7*10^{-14}$
Flux ActO ActS/ActS**	3669.7 + 718.3	5006.3 ± 1809.6	$6.5*10^{-12}$
Flux ActO ActS/ActO**	3147.1 + 528.4	4381.5 ± 1688.6	$4*10^{-14}$
Flux ActS ActO/ ActS**	3651.1 + 713.5	4992 ± 1817.7	$3.9*^{-12}$
Flux ActO ActO/ActS**	2922.8 + 479.7	4162.8 ± 1649	$1.4*10^{-14}$
Flux ActS ActS/ActO**	3431.3 + 648.6	4768.4 ± 1767.1	$2.8*10^{-13}$
Chain ActS/ActO	3150.2 + 184.1	3117.1 ± 183.4	0.20
Chain ActO/ActS	3149.3 + 186.1	3109.3 ± 169.9	0.24
Source ActS/ActO	5951.8 + 94.6	5903.1 ± 309.2	0.21
Sink ActS/ActO	5984 + 289.1	5963.1 ± 324.6	0.65
Cycle ActS ActS TfO**	588.9 + 50.4	881.60 ± 76.4	$1.6*10^{-22}$
Cycle TfO ActS ActS**	985.6 + 125.5	1348.8 ± 216.4	$1.5*10^{-18}$
Flux ActS TfO/TfO**	1895.9 + 163	2747.3 ± 229.6	$2.0*10^{-22}$
Flux TfO ActS/ActS**	2852.2 + 354.2	3990.9 ± 638.5	$1.5*10^{-19}$
Flux TfO ActS/TfO**	1874.2 + 150.9	2726.1 ± 216.9	$1.4*10^{-22}$
Flux ActS TfO/ ActS**	2877.6 + 363.2	4012.6 ± 632.7	$2.5*10^{-19}$
Flux TfO TfO/ActS**	1700.3 + 148.3	2543.8 ± 218.2	$2.*10^{-22}$
Flux ActS ActS/TfO**	2666 + 298.1	3775.4 ± 592.5	$1.4*10^{-20}$
Chain ActS/TfO	2528.4 + 166.6	2537.3 ± 135.6	0.34
Chain TfO/ActS	2522.1 + 162.2	2520 ± 129.7	0.61
Source ActS/TfO*	4709.1 + 209.1	4800.3 ± 202.9	0.007
Sink ActS/TfO*	4805.1 + 222.5	4889 ± 202.8	0.007
Cycle TfS TfS ActO**	643.6 + 76.4	901.10 ± 200.6	$7.6*10^{-19}$
Cycle ActO TfS TfS**	521.8 + 41.5	704.60 ± 70.8	$1.9*10^{-21}$
Flux TfS ActO/ActO**	1981.3 + 223.2	2739.1 ± 594.2	$1.1*10^{-18}$
Flux ActO TfS/TfS**	1552.1 + 125.9	2116.5 ± 207.9	$7.5*10^{*-22}$
Flux ActO TfS/ActO**	1972.5 + 227.2	2732.6 ± 598	$1.4*10^{-18}$
Flux TfS ActO/ TfS**	1556.6 + 125.1	2114 ± 211.7	$1.2*10^{-21}$
Flux ActO ActO/TfS**	1880.9 + 220.4	2637 ± 569	$7.3*10^{*-19}$
Flux TfS TfS/ActO**	1486.2 + 136.4	2042 ± 213	$7.5*10^{-22}$
Chain TfS/ActO**	2100.1 + 121.6	2181.8 ± 136.8	0.0001
Chain ActO/TfS**	2086.2 + 110.5	2201.4 ± 130.2	$3.9*10^{-07}$
Source TfS/ActO**	3933.4 + 236.6	4127.1 ± 247	$3.3*10^{-05}$
Sink TfS/ActO**	3993.6 + 226.4	4189 ± 255.6	$1.6*10^{-05}$
Cycle TfS TfS TfO**	387.7 + 44.2	589.70 ± 93	$4.8*10^{-18}$
Cycle TfO TfS TfS**	403.7 + 39.1	570.50 ± 90.1	$5.3*10^{-17}$
Flux TfS TfO/TfO**	1206.7 + 128.4	1794.7 ± 268.5	$3.5*10^{-18}$
Flux TfO TfS/TfS**	1220 + 115.8	1752.5 ± 260.4	$4.4*10^{-18}$
Flux TfO TfS/TfO**	1185.2 + 126.7	1780.1 ± 266	$7.9*10^{-19}$

Flux TfS TfO/ TfS**	1203.8 + 115.1	1735.5 ± 261.1	$4.6*10^{-18}$
Flux TfO TfO/TfS**	1128.8 + 143	1723.4 ± 269.4	$3.8*10^{-18}$
Flux TfS TfS/TfO**	1155.4 + 127.6	1680.1 ± 264.5	$8.5*10^{-18}$
Chain TfS/TfO**	1603.8 + 94.5	1723.8 ± 129.1	$2.5*10^{-11}$
Chain TfO/TfS**	1593.7 + 92.6	1728.5 ± 126.9	$1.2*10^{-12}$
Source TfS/TfO**	3088.6 + 192.2	3353.3 ± 252.6	$5.4*10^{-12}$
Sink TfS/TfO**	3044.9 + 198.1	3322.6 ± 264.4	$3.9*10^{-12}$
Cycle ActO ActO TfO**	471.1 + 55.6	718.40 ± 71.3	$1.3*10^{-22}$
Cycle TfO ActO ActO**	613.1 + 74.9	906.60 ± 197.8	$2.7*10^{-21}$
Flux ActO TfO/TfO**	1479.1 + 148.7	2231.4 ± 206.2	$1.2*10^{-22}$
Flux TfO ActO/ActO**	1909.1 + 219.9	2825.1 ± 595.5	$2.4*10^{-21}$
Flux TfO ActO/TfO**	1473.1 + 143.6	2229.4 ± 204.2	$9.8*10^{-23}$
Flux ActO TfO/ ActO**	1902.1 + 220.8	2808.6 ± 601	$3.7*10^{-21}$
Flux TfO TfO/ActO**	1466.9 + 153.9	2226 ± 214.4	$1.3*10^{-22}$
Flux ActO ActO/TfO**	1874.7 + 221.2	2751.5 ± 589.9	$4.5*10^{-21}$
Chain ActO/TfO**	1844.5 + 103.7	2031.1 ± 115.4	$1.2*10^{-15}$
Chain TfO/ActO**	1845.3 + 96.7	2029.5 ± 112.4	$9.1*10^{-16}$
Source ActO/TfO**	3851 + 206.8	4113.2 ± 219.1	$2.8*10^{-10}$
Sink ActO/TfO**	3828.9 + 200.1	4125.5 ± 219.2	$4.3*10^{-12}$

Table S5. **2-order motif analysis, statistical comparison.** (*) significant, (**) significant Bonferroni.

motif	ctrl	patients	p-value
Cycle ActS TfS ActO**	815.3 + 78.4	1087.4 + 186.9	7.210^{*-20}
Cycle ActS ActO TfS**	812.7 + 77.8	1092.6 + 189.9	$4.9*10^{-20}$
Flux ActS TfS/ActO**	2275.8 + 213.6	3119.8 + 544.1	$1.2*10^{-20}$
Flux ActS ActO/TfS**	2344.8 + 230.1	3173.2 + 550.7	$6.1*10^{-20}$
Flux TfS ActS/ActO**	2243.4 + 206.3	3092.5 + 544.3	$7.6*10^{-21}$
Flux TfS ActO/ActS**	2397.8 + 241.1	3238.6 + 571.9	$8.2*10^{-20}$
Flux ActO ActS/TfS**	2326.1 + 219.2	3154.6 + 541.5	$2.6*10^{-20}$
Flux ActO TfS/ActS**	2394.1 + 237	3252.2 + 571.3	$4.3*10^{-20}$
Cycle ActS TfO ActO**	758 + 70.4	1089.3 + 183.9	$1.1*10^{-21}$
Cycle ActS ActS TfO**	757.7 + 69.4	1088.8 + 187.8	$1.1*10^{-21}$
Flux ActS TfO/ActO**	2343.4 + 221	3310.2 + 561.1	$1.5*10^{-21}$
Flux ActS ActO/TfO**	2349.2 + 211.7	3324.4 + 543.7	$1.3*10^{-21}$
Flux TfO ActS/ActO**	2358.2 + 216.4	3313.7 + 572.2	$1.7*10^{-21}$
Flux TfO ActO/ActS**	2206.5 + 197.2	3175.5 + 546.7	$2.3*10^{-22}$
Flux ActO ActS/TfO**	2371.5 + 215.6	3337.3 + 544	$1.5*10^{-21}$
Flux ActO TfO/ActS**	2208.9 + 197.6	3180 + 548.6	$4.2*10^{-22}$
Cycle TfS ActS TfO**	625.4 + 38.5	867.7 + 69.30	$1.9*10^{-22}$
Cycle TfS TfO ActS**	628.2 + 38.7	865.5 + 67.40	$2.2*10^{-22}$
Flux TfS ActS/TfO**	1768.5 + 102.7	2505 + 194.3	$1.0*10^{-22}$
Flux TfS TfO/ActS**	1863.4 + 115.1	2596.4 + 204.3	$1.9*10^{-22}$
Flux ActS TfS/TfO**	1734.1 + 105.1	2474.3 + 192.8	$1.3+10^{-22}$
Flux ActS TfO/TfS**	1807.7 + 109.8	2552.7 + 203.5	$1.9*10^{-22}$
Flux TfO TfS/ActS**	1847.7 + 114.5	2590.5 + 207.7	$1.5*10^{-22}$
Flux TfO ActS/TfS**	1836.4 + 108.3	2574.1 + 204.8	$1.4*10^{-22}$
Cycle TfS ActO TfO**	497.1 + 41.4	719.5 + 64.80	$9.8*10^{-23}$
Cycle TfS TfO ActO**	497.7 + 40.3	721.1 + 64.80	$1.1*10^{-22}$
Flux TfS ActO/TfO**	1525.8 + 120.8	2182.4 + 191.9	$9.8*10^{-23}$
Flux TfS TfO/ActO**	1500.8 + 120.8	2137.1 + 193.6	$1.3*10^{-22}$
Flux ActO TfS/TfO**	1520.7 + 124.1	2184.6 + 196.1	$9.8*10^{-23}$
Flux ActO TfO/TfS**	1449.8 + 142.2	2105.3 + 199.1	$1.0*10^{-22}$
Flux TfO TfS/ActO**	1506.6 + 121.5	2146.3 + 195.2	$1.2*10^{-22}$
Flux TfO ActO/TfS**	1462.3 + 134.3	2118.5 + 195	$9.3*10^{-23}$

S5. Centrality Analysis

The centrality analysis reveals different patterns of central nodes between the groups. Tables S6 and S7 list the names of the central brain regions along with the corresponding mean and standard deviation (SD) of their strength values.

Table S6. **In- and out-strength central nodes of the HC are reported.** Central nodes are defined as those with a strength value above the 90th percentile of the strength distribution. Brain regions that appear in more than one layer of the multiplex network, corresponding to intermediate central nodes, are highlighted in bold.

ActS	TfS	ActO	TfO
Insular Cortex Right (1.22 ± 0.35)<br><br>Lingual Gyrus Left (1.23 ± 0.40)<br><br>Planum Polare Right (1.23 ± 0.42)<br><br>Planum Temporale Right (1.23 ± 0.39)<br><br>**Supracalcarine Cortex Right (1.24 ± 0.40)**<br><br>Cingulate Gyrus, anterior division (1.24 ± 0.37)<br><br>Superior Temporal Gyrus, anterior division Left (1.24 ± 0.41)<br><br>Lingual Gyrus Right (1.25 ± 0.36)<br><br>Occipital Fusiform Gyrus Left (1.26 ± 0.44)<br><br>Intracalcarine Cortex Left (1.27 ± 0.43)<br><br>Middle Temporal Gyrus, temporooccipital part Right (1.27 ± 0.38)	**Supracalcarine Cortex Right (0.47 ± 0.16)**<br><br>Frontal Operculum Cortex Left (0.47 ± 0.13)<br><br>Intracalcarine Cortex Right (0.47 ± 0.15)<br><br>Inferior Frontal Gyrus, pars triangularis Left (0.47 ± 0.18)<br><br>Inferior Temporal Gyrus, temporooccipital part Right (0.47 ± 0.17)<br><br>Inferior Temporal Gyrus, posterior division Left (0.47 ± 0.12)<br><br>Accumbens Right (0.48 ± 0.15)<br><br>Occipital Pole Left (0.47 ± 0.17)<br><br>Occipital Pole Right (0.48 ± 0.17)<br><br>**Pallidum Right (0.49 ± 0.19)**<br><br>**Pallidum Left (0.50 ± 0.17)**	Angular Gyrus Left (0.85 ± 0.30)<br><br>Angular Gyrus Right (0.86 ± 0.31)<br><br>Frontal Medial Cortex (0.86 ± 0.25)<br><br>**Pallidum Right (0.86 ± 0.43)**<br><br>**Pallidum Left (0.87 ± 0.41)**<br><br>Thalamus Left (0.89 ± 0.34)<br><br>**Putamen Left (0.89 ± 0.33)**<br><br>Putamen Right (0.90 ± 0.37)<br><br>Thalamus Right (0.92 ± 0.36)<br><br>**Caudate Left (0.92 ± 0.34)**<br><br>**Caudate Right (0.93 ± 0.34)**	Superior Parietal Lobule Left (0.47 ± 0.17)<br><br>Inferior Frontal Gyrus, pars opercularis Right (0.47 ± 0.17)<br><br>Putamen Right (0.47 ± 0.14)<br><br>Lateral Occipital Cortex, superior division Right (0.47 ± 0.16)<br><br>Precentral Gyrus Right (0.47 ± 0.18)<br><br>**Caudate Right (0.48 ± 0.16)**<br><br>Middle Temporal Gyrus, posterior division Right (0.48 ± 0.15)<br><br>Supramarginal Gyrus, anterior division Right (0.48 ± 0.15)<br><br>**Caudate Left (0.48 ± 0.15)**<br><br>**Putamen Left (0.49 ± 0.17)**<br><br>Lateral Occipital Cortex, superior division Left (0.50 ± 0.16)
Insular Cortex Right (1.20 ± 0.34)<br><br>**Hippocampus Right (1.20 ± 0.38)**<br><br>Temporal Pole Left (1.21 ± 0.34)<br><br>Heschl's Gyrus Left	Amygdala Left (0.48 ± 0.12)<br><br>**Accumbens Left** (0.48 ± 0.12)<br><br>Putamen Right (0.48 ± 0.12)<br><br>Hippocampus Left	Supramarginal Gyrus, posterior division Left (0.86 ± 0.25)<br><br>**Intracalcarine Cortex Right (0.86 ± 0.28)**<br><br>Cingulate Gyrus, posterior division	Intracalcarine Cortex Left (0.48 ± 0.15)<br><br>**Intracalcarine Cortex Right (0.48 ± 0.13)**<br><br>Occipital Pole Right (0.49 ± 0.15)

(1.23 ± 0.34)<br><br>Supplementary Motor Cortex- Right<br>(1.23 ± 0.39)<br><br>Superior Temporal Gyrus, posterior division Right<br>(1.23 ± 0.40)<br><br>Central Opercular Cortex Right<br>(1.24 ± 0.38)<br><br>Postcentral Gyrus Right<br>(1.27 ± 0.43)<br><br>Supplementary Motor Cortex- Left<br>(1.31 ± 0.41)<br><br>Precentral Gyrus Left<br>(1.33 ± 0.39)<br><br>Precentral Gyrus Right<br>(1.37 ± 0.42)	(0.48 ± 0.14)<br><br>Putamen Left<br>(0.48 ± 0.12)<br><br>Inferior Temporal Gyrus, anterior division Left<br>(0.48 ± 0.12)<br><br>**Caudate Right**<br>(0.49 ± 0.15)<br><br>**Hippocampus Right**<br>**(0.49 ± 0.13)**<br><br>**Inferior Temporal Gyrus, posterior division Right**<br>**(0.49 ± 0.13)**<br><br>Parahippocampal Gyrus, anterior division Right<br>(0.50 ± 0.13)<br><br>Temporal Fusiform Cortex, anterior division Right<br>(0.50 ± 0.11)	(0.86 ± 0.25)<br><br>Supracalcarine Cortex Left<br>(0.87 ± 0.31)<br><br>**Thalamus Right**<br>(0.88 ± 0.32)<br><br>Caudate Left<br>(0.89 ± 0.29)<br><br>Angular Gyrus Right<br>(0.89 ± 0.23)<br><br>Pallidum Left<br>(0.89 ± 0.36)<br><br>Pallidum Right<br>(0.93 ± 0.33)<br><br>**Thalamus Left**<br>(0.93 ± 0.35)<br><br>**Caudate Right**<br>(0.93 ± 0.36)	Pallidum Left<br>(0.49 ± 0.15)<br><br>**Thalamus Right**<br>(0.49 ± 0.14)<br><br>**Inferior Temporal Gyrus, posterior division Right**<br>**(0.49 ± 0.16)**<br><br>Accumbens Right<br>(0.50 ± 0.13)<br><br>**Thalamus Left**<br>(0.50 ± 0.13)<br><br>**Accumbens Left**<br>(0.50 ± 0.15)<br><br>Inferior Temporal Gyrus, posterior division Left<br>(0.50 ± 0.14)<br><br>Pallidum Right<br>(0.50 ± 0.16)

Table S7, In- and out-strength central nodes of the SCZ are reported. Central nodes are defined as those with a strength value above the 90th percentile of the strength distribution. Brain regions that appear in more than one layer of the multiplex network, corresponding to intermediate central nodes, are highlighted in bold.

ActS	TfS	ActO	TfO
Occipital Fusiform Gyrus Left (1.32 ± 0.46)<br><br>Superior Temporal Gyrus, anterior division Left (1.33 ± 0.53)<br><br>Intracalcarine Cortex Right (1.3 ± 0.44)<br><br>Lingual Gyrus Left (1.34 ± 0.47)<br><br>**Occipital Pole Right (1.35 ± 0.40)**<br><br>Supracalcarine Cortex Right (1.35 ± 0.42)<br><br>Inferior Temporal Gyrus, temporooccipital part Right (1.36 ± 0.49)<br><br>**Occipital Fusiform Gyrus Right (1.36 ± 0.51)**<br><br>Lingual Gyrus Right (1.38 ± 0.49)<br><br>Temporal Pole Right (1.38 ± 0.43)<br><br>Cuneus Right (1.41 ± 0.46)	Inferior Temporal Gyrus, anterior division Left (0.51 ± 0.12)<br><br>Amygdala Right (0.51 ± 0.14)<br><br>**Occipital Fusiform Gyrus Right (0.51 ± 0.14)**<br><br>Occipital Pole Left (0.51 ± 0.17)<br><br>Precuneous Cortex (0.51 ± 0.17)<br><br>Cingulate Gyrus, posterior division (0.52 ± 0.13)<br><br>Thalamus Right (0.52 ± 0.15)<br><br>Inferior Temporal Gyrus, posterior division Right (0.52 ± 0.14)<br><br>Supracalcarine Cortex Right (0.52 ± 0.13)<br><br>Supracalcarine Cortex Left (0.52 ± 0.14)<br><br>**Occipital Pole Right (0.54 ± 0.17)**	Pallidum Left (0.98 ± 0.42)<br><br>Hippocampus Right (0.99 ± 0.39)<br><br>Frontal Operculum Cortex Right (1.00 ± 0.34)<br><br>Caudate Right (1.00 ± 0.41)<br><br>Middle Temporal Gyrus, anterior division Right (1.02 ±0.37)<br><br>Hippocampus Left (1.02 ± 0.41)<br><br>Frontal Operculum Cortex Left (1.03 ± 0.47)<br><br>Subcallosal Cortex (1.05 ± 0.42)<br><br>Putamen Right (1.05 ± 0.35)<br><br>Caudate Left (1.05 ± 0.48)<br><br>Putamen Left (1.13 ± 0.43)	Superior Frontal Gyrus Right (0.53 ± 0.16)<br><br>Precentral Gyrus Right (0.53 ± 0.17)<br><br>Parahippocampal Gyrus, anterior division Right (0.53 ± 0.18)<br><br>Parahippocampal Gyrus, posterior division Left (0.53 ± 0.17)<br><br>Supplementary Motor Cortex- Right (0.53 ± 0.1400)<br><br>Caudate Right (0.54 ± 0.15)<br><br>Hippocampus Right (0.54 ± 0.19)<br><br>Parahippocampal Gyrus, anterior division Left (0.55 ± 0.17)<br><br>Caudate Left (0.56 ± 0.15)<br><br>Putamen Left (0.56 ± 0.15)<br><br>Putamen Right (0.57 ± 0.16)
Parietal Operculum Cortex Right (1.37 ± 0.46)<br><br>Planum Temporale Right (1.37 ± 0.53)<br><br>Insular Cortex Left (1.38 ± 0.44)	Parahippocampal Gyrus, anterior division Right (0.51 ± 0.12)<br><br>Frontal Orbital Cortex Right (0.52 ± 0.14)	Middle Frontal Gyrus Left (0.97 ± 0.42)<br><br>Occipital Pole Right (0.98 ± 0.39)<br><br>Frontal Medial Cortex (0.98 ± 0.34)	Supracalcarine Cortex Right (0.53 ± 0.16)<br><br>Temporal Fusiform Cortex, anterior division Right (0.53 ± 0.17)

**Putamen Left**<br>**(1.39 ± 0.47)**<br><br>Central Opercular Cortex Left<br>(1.39 ± 0.40)<br><br>Central Opercular Cortex Right<br>(1.42 ± 0.42)<br><br>Insular Cortex Right<br>(1.43 ± 0.49)<br><br>Precentral Gyrus Right<br>(1.43 ± 0.51)<br><br>Supplementary Motor Cortex- Left<br>(1.44 ± 0.49)<br><br>Supplementary Motor Cortex- Right<br>(1.44 ± 0.43)<br><br>Precentral Gyrus Left<br>(1.45 ± 0.46)	Temporal Fusiform Cortex, anterior division Right<br>(0.53 ± 0.14)<br><br>Caudate Left<br>(0.53 ± 0.17)<br><br>**Pallidum Right**<br>**(0.53 ± 0.17)**<br><br>Accumbens Left<br>(0.53 ± 0.13)<br><br>Caudate Right<br>(0.53 ± 0.15)<br><br>Subcallosal Cortex<br>(0.54 ± 0.14)<br><br>Pallidum Left<br>(0.54 ± 0.13)<br><br>Putamen Right<br>(0.55 ± 0.14)<br><br>**Putamen Left**<br>**(0.58 ± 0.17)**	Angular Gyrus Left<br>(0.98 ± 0.41)<br><br>Middle Frontal Gyrus Right<br>(0.98 ± 0.37)<br><br>Inferior Temporal Gyrus, posterior division Right<br>(0.98 ± 0.41)<br><br>Supramarginal Gyrus, posterior division Right<br>(0.98 ± 0.47)<br><br>Inferior Temporal Gyrus, temporooccipital part Left<br>(0.98 ± 0.42)<br><br>Cingulate Gyrus, posterior division<br>(1.01 ± 0.35)<br><br>Angular Gyrus Right<br>(1.01 ± 0.48)<br><br>Precuneous Cortex<br>(1.03 ± 0.43)	Inferior Temporal Gyrus, temporooccipital part Left<br>(0.53 ± 0.18)<br><br>**Pallidum Right**<br>**(0.53 ± 0.14)**<br><br>Amygdala Left<br>(0.53 ± 0.15)<br><br>Middle Frontal Gyrus Left<br>(0.54 ± 0.19)<br><br>Frontal Pole Left<br>(0.54 ± 0.17)<br><br>Inferior Temporal Gyrus, posterior division Right<br>(0.54 ± 0.15)<br><br>Accumbens Right<br>(0.55 ± 0.15)<br><br>Occipital Pole Right<br>(0.55 ± 0.16)

References

1. Desikan, R.S.; Ségonne, F.; Fischl, B.; Quinn, B.T.; Dickerson, B.C.; Blacker, D.; Buckner, R.L.; Dale, A.M.; Maguire, R.P.; Hyman, B.T.; Albert, M.S.; Killiany, R.J. An automated labeling system for subdividing the human cerebral cortex on MRI scans into gyral based regions of interest. *Neuroimage* **2006,** 31(3), 968–80.
2. Parente, F.; Colosimo, A. Modelling a multiplex brain network by local transfer entropy. *Sci. Rep.* **2021**, *11*(11), 15525.
